# Interrelation between Stress Management and Secretion Systems of *Ralstonia solanacearum*: An In Silico Assessment

**DOI:** 10.3390/pathogens11070730

**Published:** 2022-06-27

**Authors:** Goutam Banerjee, Fu-Shi Quan, Amit Kumar Mondal, Shantanu Sur, Pratik Banerjee, Pritam Chattopadhyay

**Affiliations:** 1 Department of Food Science and Human Nutrition, University of Illinois at Urbana-Champaign, Urbana, IL 61801, USA; goutamb@illinois.edu; 2 Department of Medical Zoology, Kyung Hee University School of Medicine, Seoul 02447, Korea; fquan01@gmail.com; 3 Medical Research Center for Bioreaction to Reactive Oxygen Species and Biomedical Science Institute, School of Medicine, Graduate School, Kyung Hee University, Seoul 02447, Korea; 4 Department of Biological Sciences, Texas Tech University, Lubbock, TX 79409, USA; amitvb677@gmail.com; 5 Department of Biology, Clarkson University, Potsdam, NY 13699, USA; ssur@clarkson.edu; 6 Department of Botany, M.U.C. Women’s College, University of Burdwan, Bardhaman 713104, West Bengal, India

**Keywords:** host-mediated stress, bacterial stress management, protein–protein interaction, T3SS, GSP, pili

## Abstract

*Ralstonia solanacearum* (Rs), the causative agent of devastating wilt disease in several major and minor economic crops, is considered one of the most destructive bacterial plant pathogens. However, the mechanism(s) by which Rs counteracts host-associated environmental stress is still not clearly elucidated. To investigate possible stress management mechanisms, orthologs of stress-responsive genes in the Rs genome were searched using a reference set of known genes. The genome BLAST approach was used to find the distributions of these orthologs within different Rs strains. BLAST results were first confirmed from the KEGG Genome database and then reconfirmed at the protein level from the UniProt database. The distribution pattern of these stress-responsive factors was explored through multivariate analysis and STRING analysis. STRING analysis of stress-responsive genes in connection with different secretion systems of Rs was also performed. Initially, a total of 28 stress-responsive genes of Rs were confirmed in this study. STRING analysis revealed an additional 7 stress-responsive factors of Rs, leading to the discovery of a total of 35 stress-responsive genes. The segregation pattern of these 35 genes across 110 Rs genomes was found to be almost homogeneous. Increasing interactions of Rs stress factors were observed in six distinct clusters, suggesting six different types of stress responses: membrane stress response (MSR), osmotic stress response (OSR), oxidative stress response (OxSR), nitrosative stress response (NxSR), and DNA damage stress response (DdSR). Moreover, a strong network of these stress responses was observed with type 3 secretion system (T3SS), general secretory proteins (GSPs), and different types of pili (T4P, Tad, and Tat). To the best of our knowledge, this is the first report on overall stress response management by Rs and the potential connection with secretion systems.

## 1. Introduction

*Ralstonia solanacearum* (Rs) is a group of aerobic, non-spore-forming, Gram-negative plant pathogens that belong to the Burkholderiaceae family of the β-proteo-bacteria group. Rs is a “bacterial species complex” consisting of four monophyletic phylotypes [1]. This pathogen is designated as the world’s deadliest bacterial plant pathogen because of its wide-range host specificity (>200 plant species), broad geographical distribution, abundance and persistence in soil, root invasion, tissue-specific tropism, multiplication, and colonization (>10^9^ C.F.U. per g fresh weight) in xylem vessels [2,3]. Rs is also considered “quarantine organisms”, “bioterrorism”, and “double usage agents” by different regulatory authorities in the USA and Europe [4]. On the other hand, due to wide host diversity, Rs may be an attractive model to investigate the question of adaptation to the host environment. 

The stress response in bacteria involves processes that enable bacteria to survive in adverse environmental conditions. The genetic makeup helps bacteria to sense the changing environment and to react accordingly by modulating the expression of certain gene cascades and protein activities. In particular, stress response management is critical for pathogens to survive inside the host and to defeat the host’s primary immunity. The stress response in bacteria is a multi-network phenomenon, and different sets of genes responsible for different types of stresses (such as acidic, alkaline, desiccation, osmotic, nutrient deficiency, heat, cold, etc.) are expressed accordingly with the help of effector molecules to maintain cellular integrity [5]. According to Hengge-Aronis (2002), the bacterial stress response can be defined as the sequential alteration of the expression of several genes and their protein levels to cope with extreme conditions [6]. Like all other pathogenic bacteria, Rs also must face stresses during the first physical contact with the host. It is already well known that plant roots secrete reactive oxygen species (ROS) for their own protection in the rhizosphere environment, which may impart host-associated stress to Rs during the first interaction with the plant [7,8]. Inside the host, different types of signals (e.g., pH and temperature) and nutrient deficiency accelerate the adaptive stress responses in Rs, along with the expression of virulence genes. For example, the Dps gene was reported to contribute to oxidative stress tolerance during tomato plant infection by Rs [7]. Flores-Cruz and Allen (2011) reported several ROS-scavenging enzymes and the OxyR gene in the genome of Rs, which play a critical role during stress conditions or exposure to extreme environments [9]. Fang et al. (2016) classified bacterial stress responses during their infection of hosts; however, the detailed mechanism of stress management in Rs has not been fully explored to date. Notably, the correlation between stress responses and the secretion systems of Rs needs a deeper understanding.

The identification and distribution of Rs proteins responsible for regulating resistance to different types of host-environment stress conditions could be important factors in pathogenicity. In the present investigation, an in silico approach was taken to identify and monitor the distribution of stress-responsive genes and their relationship with bacterial secretion systems of Rs.

## 2. Results

### 2.1. Mining and Categorization of Rs Stress-Responsive Genes

Based on experimental records, the initial number of query candidates for bacterial stress-responsive genes (SRGs) was 47 (Table 1).

However, based on orthologs present in Rs, the numbers were reduced to 28 (Table S1). The protein sequences of these 28 SRGs were then used as a secondary query for pBLAST across available annotated Rs genomes (Table S2). A protein–protein interaction network (PIN) was studied within Rs genomes using STRING V11, taking these 28 SRGs as an input file. In string analysis, increasing interactions (no. of edges expected: 39; found: 88) resulted in a total no. of 35 nodes (representing genes, average node degree: 5.03), which indicated 35 SRGs within Rs (Table 2). Thus, in addition to the initially identified 28 SRGs, 7 new SRGs (total of 35 SRGs) were discovered from STRING analysis (Table S3).

**Figure 1:**
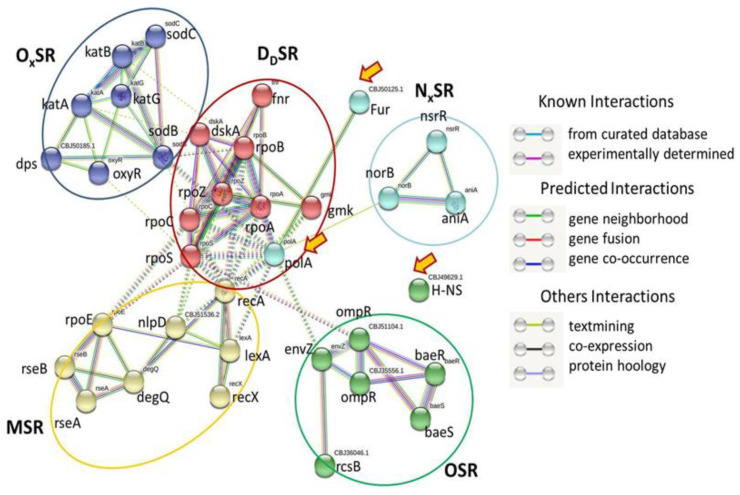
STRING analysis results showing protein–protein interaction network in stress-responsive genes of *R. solanacerium*.

The cluster analysis (k-means clustering) of these 35 SRGs revealed five distinct clusters indicating five different types of stress responses: membrane stress response (MSR), osmotic stress response (OSR), oxidative stress response (OxSR), nitrosative stress response (NxSR), and DNA damage stress response (DdSR) (Figure 1).

However, a PIN was not observed in the case of Histone-like protein H-NS (ortholog of K03746, BBJ49629.1), associated with temperature-dependent stress regulation. Interestingly, DdSR was found in the center among these five clusters, showing independent PIN connections with the other four clusters.

#### 2.1.1. MSR

The present analysis revealed that eight SRGs (including nlpD ortholog CBJ51536.2) contributed to the MSR cluster (Figure 1). The cluster is composed of outer membrane metallopeptidase (NlpD), periplasmic serine endoprotease (DegQ), RNA polymerase σ (RpoE), transcription factor (LexA) repressor, and binary systems (RseA and RseB; RecA and RecX). In Figure 1, the PIN between alternative σE (RpoE and RseA), degQ, and two-component systems Rcs (RcsB and RcsC) is distinct. The MSR cluster is linked to the other four clusters via the DdSR cluster. In particular, a PIN was found among rpoE, degQ, nlpD, recA, and lexA of MSR with rpoA, rpoB, rpoC, and rpoS of DdSR.

#### 2.1.2. OSR

Six SRG candidates (including ompR orthologs CBJ35556.1 and CBJ51104.1) were found to construct the cluster OSR (Figure 1). In the present investigation, a strong PIN was recorded among the osmotic stress regulator ompR and envZ of Rs. Along with ompR, a strong PIN was also connected to the two-component system Bae (BaeR and BaeS). The OSR cluster relates to the DdSR cluster via ompR and envZ.

#### 2.1.3. OxSR 

The present analysis revealed that seven SRGs (including Dps ortholog CBJ50185.1) contributed to the OxSR cluster (Figure 1). Three orthologs of the enzyme catalase (katA, katB, and katG) and two orthologs of the enzyme superoxide dismutase (sodB and sodC) were identified within Rs genomes (Table 2). Besides multiple and redundant ROS-scavenging enzymes, PINs within oxidative stress regulators such as Dps and oxyR were also identified (Figure 1). In Figure 1, it appears that KatA, KatB, KatG, SodB, and SodC work together in an interactive fashion to provide protection against oxidative stress. The carbon and nitrogen starvation regulator Dps (Figure 1) interacts with stress-responsive enzymes (katE and sodB) and another stress regulator (oxyR). String analysis also suggests that oxyR also interacts with other stress-responsive enzymes (katE and sodB) and stress regulators (Dps) (Figure 1). The PIN between the OxSR cluster and the DdSR cluster is mediated via sodB, kata, katB, and oxyR.

#### 2.1.4. NxSR

Three SRG candidates (nsrR, norB, and aniA) were found to construct the cluster NxSR (Figure 1). Two probable candidates (polA and fur ortholog CBJ50125.1) were not included in this cluster due to the lack of evidence (known interactions) of the involvement of these two SRGs in the nitrosative stress response. A single ortholog (norB) was identified for the enzyme nitric-oxide reductase (Table 2). The PIN obtained from the STRING server indicates interactions among these three SRG candidates (Figure 1). However, a single PIN was observed between the clusters NxSR and DdSR, mediated through norB.

#### 2.1.5. DdSR

The present investigation discovered eight to ten (including polA and fur ortholog CBJ50125.1) SRG candidates that constitute the DdSR cluster (Figure 1). This cluster is composed of DNA polymerase I (polA), different RNA polymerase subunits (rpoA for α, rpoC for β, rpoS for σ, and rpoZ for ω), transcription factors (dskA and fnr), and kinase (gmk). RpoC is a known amino acid starvation regulator, and Fur is a known metal stress regulator, whereas FNR is considered an oxygen sensor. The DdSR cluster is the central cluster of all SRG candidates, and all other clusters are independently linked to it via PIN.

### 2.2. Homogeneous Distribution of SRGs across Rs Genomes

**Figure 2:**
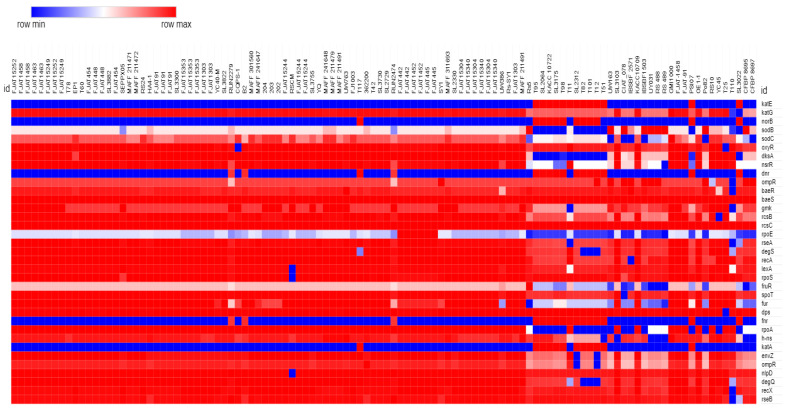
Heatmap showing the distribution of stress-responsive genes among *R. solanacerium* genomes. Row min = 0; row max = 1.

Among 35 identified SRGs (Table 2), 27 were found to be present in 110 fully annotated Rs genomes taken from the NCBI database. The heatmap analysis with the BLAST results of these 35 SRGs also indicates the same (Figure 2).

We also constructed a cluster dendrogram of the 110 experimental Rs genomes based on the presence and absence of SRGs identified in the present study. We observed most of the strains (92) to be on a straight line (Figure S1), indicating no significant differences in the pattern of stress responses among the different strains of Rs.

### 2.3. SRGs and Secretion Systems of Rs

For secretion system components, we used the KO numbers published in our previous study with *Bradyrhizobium* spp. [48] as reference data, and the results are provided in Table S4. String analysis of stress-responsive genes in relation to different secretion systems (type secretion systems including T1SS, T2SS, T3SS, T4SS, T5SS, and T6SS; general secretory protein system or GSPS; and pili systems including T4P, Tad, and Tat) of Rs was performed. A strong PIN with SRGs was found for three different secretion systems, viz. T3SS, GSPS, and pili systems (T4P, Tad, and Tat).

### 2.4. SRGs and T3SS

**Figure 3:**
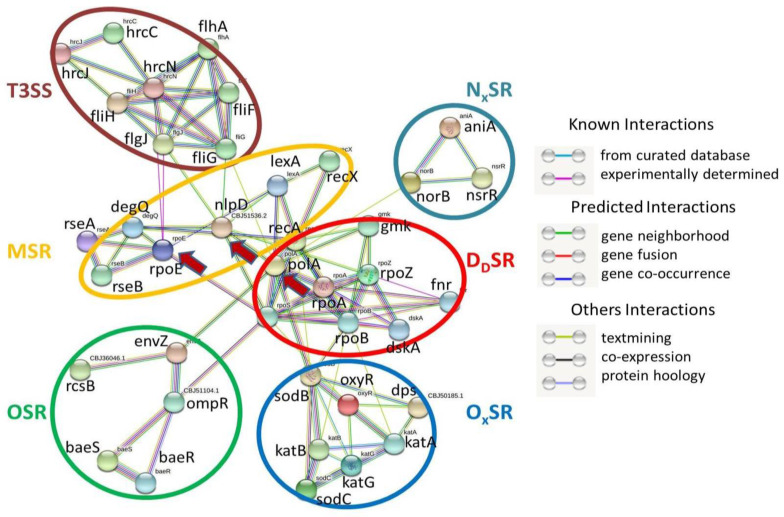
STRING analysis results showing protein–protein interaction network within stress-responsive genes and type III secretion system (T3SS) of *R. solanacerium*.

Initially, all 35 SRGs (found in this study, Table 2) and 14 T3SS candidate genes (from our previous study) were used as the input file for the String analysis. However, for a better understanding of the PIN between SRGs and T3SS, the input file was manually curated. The final output file (no. of edges expected: 39; found: 99) resulted in a total no. of 39 nodes (representing genes, average node degree: 5.08). Among these 39 nodes, 8 represent T3SS candidate genes (hrcC, hrcJ, hrcN, fliF, fliG, fliH, flhA, and flgJ) and are present as a single cluster (Figure 3).

**Figure 4:**
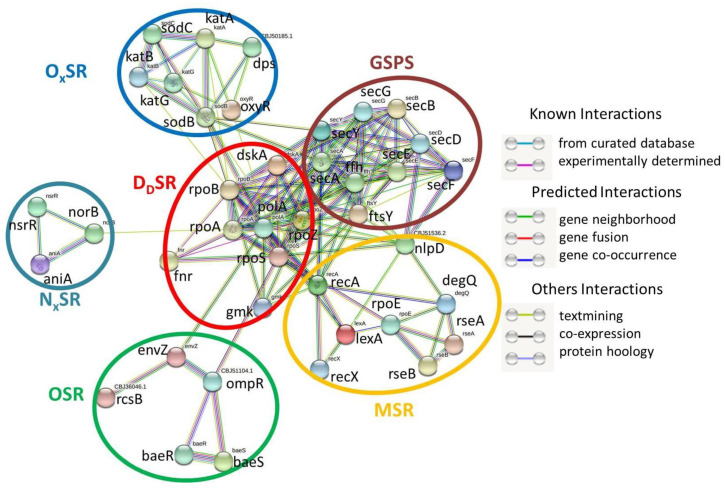
STRING analysis results showing protein–protein interaction network within stress-responsive genes and general secretory protein system (GSPS or Sec system) of *R. solanacerium*.

The candidates for the T3SS cluster are provided in Table S4. In Figure 4, out of 35 SRGs (except for h-ns, fur, ompR, and dnr), 31 were found to be distributed into the five predetermined clusters (Figure 3). However, a PIN was found to be present between T3SS (via flgJ and fliG) and MSR (via rpoE and nlpD) and DdSR (via polA) clusters of SRGs (Figure 3).

### 2.5. SRGs and GSPS

At first, all 35 SRGs (found in this study, Table 2) and 9 GSPS candidate genes (from our previous study) were used as an input file for the String analysis. The final output file (no. of edges expected: 54; found: 146) resulted in a total no. of 40 nodes (representing genes, average node degree: 7.3). Among these 40 nodes, 9 represent GSPS candidate genes (secA, secB, secD, secE, sceF, secG, secY, ftsY, and ffh) and are present as a single cluster (Figure 4). Information on the candidate genes in the GSPS cluster is provided in Table S4.

Among 35 SRGs (except for h-ns, fur, ompR, and dnr), 31 were distributed into the five predetermined clusters (Figure 4). There was no direct PIN between GSPS and DdSR, GSPS and NxSR, and GSPS and OSR. In contrast, a PIN was recorded between the GSPS cluster (most of the candidate genes) and the MSR cluster (via nlpD and recA). A PIN was also found to exist between the GSPS cluster (via secA and secV) and the OxSR cluster (via sodB and oxyR). A strong PIN was also observed between the GSPS cluster (most of the candidate genes) and almost all SRGs of the DdSR cluster (except fnr). 

### 2.6. SRGs and Pili Systems

**Figure 5:**
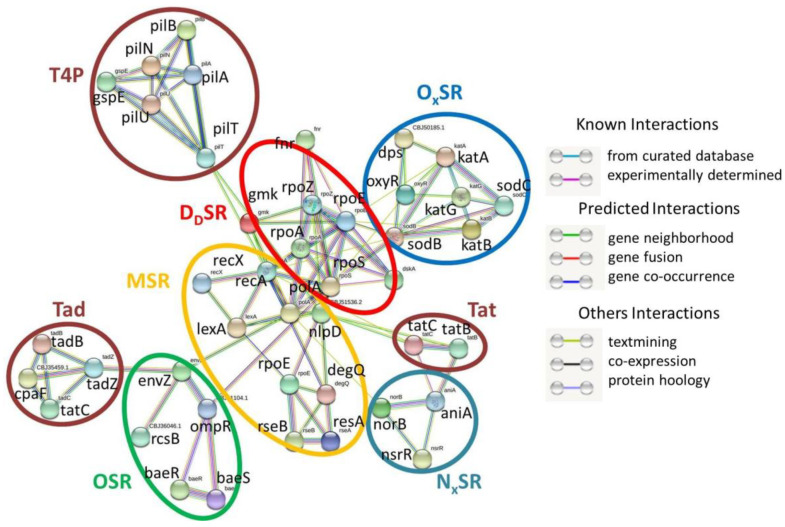
STRING analysis results showing protein–protein interaction network within stress-responsive genes and multiple (T4P, Tat, and Tad) pili systems of *R. solanacerium*.

Initially, all 35 SRGs (found in Table 2) and 26 pili system candidate genes (from our previous study) were used as input files for the String analysis. The output file (no. of edges expected: 38; found: 104) contained a total no. of 43 nodes representing genes with an average node degree of 4.84 (Figure 5).

Among these 43 nodes, 12 are represented by different pili candidate genes and are present as three separate clusters representing three different pili systems: type 4 pili or T4P (pilA, pilB, pilN, pilT, pilU, and gspE), tat pili (tatB and tatC), and tad pili (tadB, tadC, tadZ, and cpaF) (Figure 5). The SRGs were distributed into five clusters as before (Figure 5). A PIN was observed between T4P (via pilT) and DdSR (via gmk) and MSR (via recA), between Tat (via tatC and tatD) and MSR (via nlpD), DdSR (via recA), and NxSR (via aniA), and between Tad (via tadZ) and OSR (via envZ).

## 3. Discussion

### 3.1. MSR SRGs of Rs

In the present study, we observed that protein–protein interactions between σE and two-component systems such as Bae and Rcs were distinct in Rs. We report the interaction with DegQ (Table 2), a type 1 protease responsible for cleaving RseA and releasing σE/rpoE. The most crucial component of membrane stress, σE is extensively studied in human pathogens and is known to bind with the cytoplasmic membrane with the help of RseA [49]. The RecA- and LexA-guided SOS response is a well-studied phenomenon in several human enteric pathogens [19]. LexA is also reported to control the virulence gene expression in pathogenic bacteria [20]. Membrane permeability is affected by lexA and recA mutations in *Escherichia coli* K12 [50]. Genotoxic agents (e.g., mitomycin C) can significantly increase membrane vesicle production by promoting RecA-dependent stress responses [51]. Graphene oxide was reported to induce MSR in Rs through a similar mechanism [52].

### 3.2. OSR SRGs of Rs

Membrane stress is generally caused by a change in osmotic pressure, low pH, exposure to antimicrobial cationic peptides, and misfolding of envelope proteins such as secretin. Rs may survive in pure water and grow at a water activity (a_W_) near 1 [53]. Evidence suggests that regulating cytoplasmic composition and hydration is a key objective of cellular homeostasis in such conditions [54]. OmpR is a crucial factor for overcoming osmotic stress while entering the host and controlling the Bae two-component systems (Table 2). MacIntyre et al. showed that the disaccharide trehalose contributes to OSR of *R. solanacearum* strain GMI1000 [55]. Rs mutants that were unable to synthesize trehalose suffered significant growth inhibition in the presence of ionic (NaCl) or nonionic (high–molecular weight polyethylene glycol) osmotic stress [55].

### 3.3. OxSR SRGs of Rs

Disruption of the membrane releases components of the electron transport chain named quinones and flavoproteins, which generate reactive oxygen species (ROS) from molecular oxygen [49]. In the present investigation, along with molecular oxygen scavenging enzymes such as catalase and superoxide dismutase, OxyR was also reported (Table 2). Flores-Cruz and Allen previously reported the crucial role of OxyR as an oxidative stress response regulator in Rs [9]. The cysteine residues in the OxyR protein undergo redox conformational changes and can sense different components of stress factors [10]. Similar to *E. coli*, the expression of Dps in Rs was reported to be increased during the stationary phase and could be mediated through OxSR [7]. Vinegar residue substrate was reported to inhibit oxidative damage in *R. solanacearum* HB511 via the regulation of excess ROS production [56]. Recently, Yang et al. reported that OxSR in *R. solanacearum* CQPS-1 induced by daphnetin is mediated by SRGs, such as katGb, coxM, Rsp0993, Rsc2493, and dnaK [57].

### 3.4. NxSR SRGs of Rs

Disruption of the membrane releases components of the electron transport chain (quinones and flavoproteins). These components are known to generate reactive nitrogen species (RNS), such as peroxynitrite (ONOO^−^) and nitric oxide (NO). RNS are very toxic to any kind of cell [58]. In this study, both ROS-scavenging enzyme nitric-oxide reductase (coded by NorB) and nitrosative stress-responsive factors (NsrR and AniA) were reported in the Rs genomes under investigation (Table 2). A recent study elucidated the nitrosative stress response in *R. solanacearum* GMI1000 and reported that NorA, HmpX, and NorB play a critical role in reducing nitrosative stress during plant pathogenesis [59].

### 3.5. DdSR SRGs of Rs

ROS, RNS, and low pH are known causal factors of DNA damage [60,61]. The σ^S^ factor and DksA are responsible for overcoming amino acid limitations by activating several amino acid biosynthesis genes [18,62]. Brown and Allen reported the involvement of three SRGs, *uvrA1* (*ipx*64), *dinF* (*ipx*62), and *dnaN* (*ipx*61), in the SOS response of *R. solanacearum* K60 against plant-derived DNA-damaging substances [63].

### 3.6. Global Strategy in Stress Response of Rs

The versatility of the pathogenicity of Rs depends on the genetic diversity [64]. The diversity of Rs is broad, and thus, the genetic variation among different phylotypes is distinct. The cluster analysis of SRGs demonstrated that there are no significant differences among the Rs species complex (Figure S1). To explain these mutually incompatible results, it is important to understand the mosaic structure of Rs genomes. Due to remarkable genetic diversity, the genomes of Rs possess a number of similar regions scattered within variable regions [64]. Usually, genes encoding stress-responsive enzymes and factors are subject to strong levels of purifying selection, similar to most housekeeping genes [65]. The present findings suggest that the stress-responsive genes in Rs are under pressure of purifying selection. Our present finding is also supported by the experimental evidence provided by Puigvert et al. [66]. Transcriptomic analysis of *R. solanacearum* UY031 in their research found only two differentially expressed genes between resistant and susceptible plant accessions, indicating that the bacterial component plays a minor role in the establishment of disease [66].

**Figure 6:**
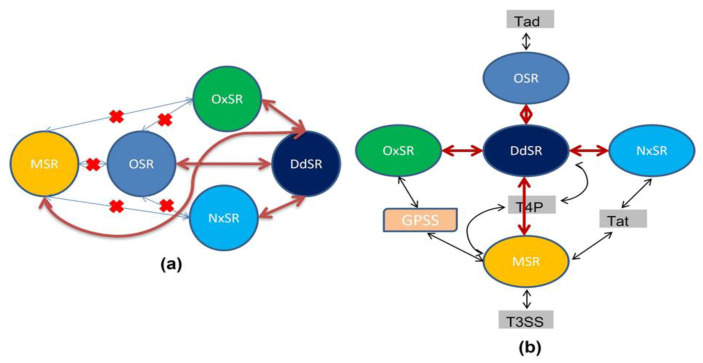
(**a**) Diagram showing interactions among clusters of stress-responsive genes (SRGs) of *R. solanacerium*: MSR (membrane stress response), OSR (osmotic stress response), OxSR (oxidative stress response), NxSR (nitrosative stress response), and DdSR (DNA damage stress response); thin lines with “X” show the absence of interactions; thick lines show the presence of interactions. (**b**) Diagram showing interactions of clusters of stress-responsive genes (SRGs) with the secretion systems of *R. solanacerium*: Tad (tight adherence), Tat (twin arginine translocation), T4P (type-4 pili), T3SS (type-3 secretion system), and GPSS (general protein secretion system); thick lines show interactions among SRGs clusters; thin lines depict the interaction between SRGs and secretion system.

Rs enters its plant host through root lesions, followed by multiplication and colonization in the root cortex and xylem, respectively. Finally, wilt symptoms develop, and then the plants die, releasing the pathogen back into the soil. From the five consistent clusters of SRGs in Rs, the stress responses can be classified into five categories (Figure 6a). Studying biological networks enables a deeper investigation of biological systems [67], and PINs have been constructed in many bacteria [68,69]. In the present study, our analysis indicates that SRGs work together interactively to fight against diverse stresses (Figure 6b). Currently, very few reports explore the relationship between clusters of SRGs and bacterial secretion systems. SRGs of the DdSR cluster were found to play a central role among all five clusters (Figure 6a). SRGs of all other clusters are linked to DdSR via PINs; however, PINs were completely absent between these other clusters. This finding strongly suggests that all other stress responses (i.e., MSR, OSR, OxSR, and NxSR) act through DdSR (Figure 6a). It also implies that the expression of SRGs of a single cluster cannot be treated as a signature of progressive stress responses.

### 3.7. Interactions among T3SS and SRGs of Rs

In the present investigation, PINs were observed between the T3SS cluster (representing eight genes: hrcC, hrcJ, hrcN, fliF, fliG, fliH, flhA, and flgJ) and the MSR cluster of SRGs (Figure 6b). Flores-Kim et al. previously reported the interaction between T3SS and MSR in Yersinia [66], which aligns with our findings. The secretion systems of Rs form a molecular arsenal that facilitates niche adaptation, host invasion, and evasion of plant defenses. As for many bacterial pathogens, the main virulence determinant in Rs is T3SS [70], which injects a number of effector proteins into plant cells, causing disease in hosts or a hypersensitive response in resistant plants. In this work, the PIN between the MSR cluster of SRGs and T3SS indicates the critical role of SRGs in infection or disease establishment (Figure 3).

### 3.8. Interactions among GSPS and SRGs of Rs

Similarly, PINs were also recorded among the GSPS cluster (representing nine genes: secA, secB, secD, secE, sceF, secG, secY, ftsY, and ffh), MSR cluster, and OxSR cluster. Interestingly, interactions between Sec and MSR in *Escherichia coli* and the Sec secretion system and OxSR in *Pseudomonas aeruginosa* were also reported [71,72]. As in most bacteria, the Sec pathway is the main route by which secretory proteins are exported across the cytoplasmic membrane in Rs [73]. Upon their exit from the membrane translocase complex, the proteins must acquire a properly folded conformation to gain full activity. Heat stress and overproduction of specific secretory proteins can result in the accumulation of misfolded proteins outside the cytoplasm [74]. The PIN between the DdSR cluster of SRGs and GSPS, along with the PIN between the MSR cluster of SRGs and GSPS, clearly indicates the importance of SRGs in the regulation of GSPS (Figure 4). The accumulation of misfolded proteins produces cellular stress responses leading to refolding or degradation of abnormally folded, non-functional proteins [75]. Rs may sense the secretion stress via the extracytoplasmic sigma factor σ^E^.

### 3.9. Interactions among Different Pili Systems and SRGs of Rs

Furthermore, we explored the interactions among the T4P cluster, MSR, and DdSR, which was reported by a previous study with *E. coli* [76]. A strong PIN was observed between the T4P cluster, the MSR cluster, and NxSR. Rs binds to host cells by a multitude of pili. Therefore, the adhesion strength of a multipili-binding bacterium largely depends on the cooperativity of the attaching pili [73]. Interestingly, different types of pili of Rs were found to be under the control of different SRG clusters. For example, T4P pili are under the control of DdSR and MSR clusters, and Tat pili are controlled by MSR and NxSR clusters, while Tad pili are under the control of the OSR cluster (Figure 6b).

### 3.10. Network of SRGs and Bacterial Secretion Systems

In this work, we show the interaction of various SRG clusters with specific secretion components. For example, T3SS, GSPs, and T4P are related to MSR and DdSR, while Tad and Tad interact with OSR and NxSR, respectively (Figure 6b). A detailed transcriptome analysis of Rs provides the experimental proof that genes encoding T3SS (hrpY, hrpX, hrpK, and hrcT) and T4P (pilG, pilH, pilN, pilM, pilY, pilW, and fimV) are upregulated during host infection, along with peroxidases, catalases (katE and katG), and alkyl hydroperoxide reductases (ahpC1 and ahpF), suggesting adaptations to combat stress during plant infection [66]. Recently, Hendrich et al. also provided experimental proof that NxSR regulates T3SS in Rs [77]. Taken together, these findings indicate a close connection between bacterial stress management and secretion systems.

## 4. Materials and Methods

### 4.1. Data Mining

Completely assembled and annotated genomes (as of 31 March 2022) of Rs were downloaded either from the NCBI genome database (https://www.ncbi.nlm.nih.gov/genome) (accessed on 31 March 2022) or from the KEGG genome database (https://www.genome.jp/kegg/genome) (accessed on 31 March 2022), which includes 110 genomes (Table S4). Sequences of stress-responsive enzymes and stress regulators were retrieved from the UniPort database. 

### 4.2. Mining of Bacterial Genes Coding for Stress-Responsive Enzymes and Stress Regulators

**Figure 7:**
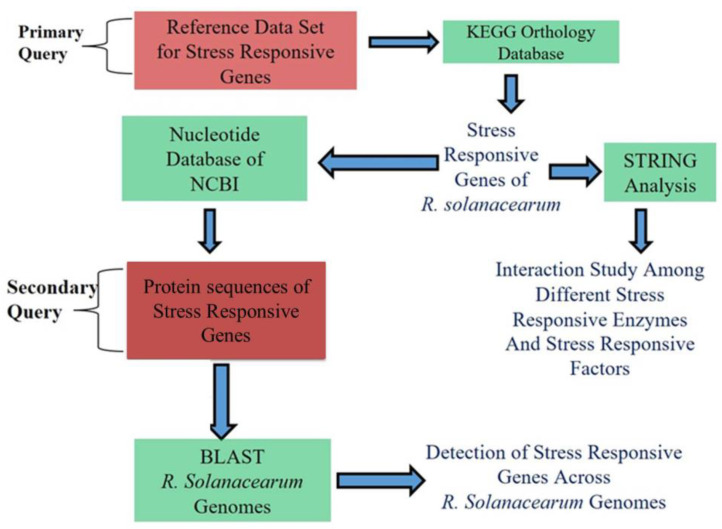
Schematic diagram of the study protocol.

The present study examined a protocol for capturing the maximum number of candidate genes associated with bacterial stress responses (Figure 7).

To develop this protocol, information regarding bacterial stress-responsive enzymes and stress regulators was collected from published articles, followed by the preparation of a dataset. This dataset was used as a primary reference query in subsequent steps (Table 1).

Initially, the presence of these candidate genes within Rs genomes was searched using the KEGG platform, and orthologs were identified from the KO database of the KEGG platform (Table 2).

Nucleotide sequences of KO orthologs of the SRGs are presented in Table S1. The secondary query was carried out on Rs genomes using KO ortholog protein sequences. To examine the diversity of these genes across Rs genomes, microbial genome BLASTs were performed using protein sequences and the megaBLAST algorithm (Tables S1 and S2). Furthermore, the results obtained from the pBLAST search were again verified with the help of the KO orthology database in KEGG among the available Rs genomes.

### 4.3. Mining of Bacterial Secretion System Genes

For secretion system components, we used the KO numbers published in our previous study with *Bradyrhizobium* spp. [48] as reference data, and the results are provided in Table S4.

### 4.4. Protein–Protein Interaction Network (PIN) Study

PINs are an important clue for understanding the cellular physiological process, and STRING is one of the best platforms to construct and visualize such interaction networks [78]. The STRING v11.0 (http://string-db.org) (accessed on 22 May 2022) server is used for predicting structure–function aspects of genes and proteins [79]. Based on the approximate probability that a predicted link exists between two proteins in the same metabolic map in the KEGG database, different ranges of confidence scores of protein–protein interactions were assigned. The SRG sequences of Rs were aligned with Rs PS107 using BLASTX. The first aligned protein with an *E*-value below 1 × E^−10^ was considered a homologous protein. Then, these homologous proteins and their corresponding interactions were extracted from the whole interaction dataset of the related organism to compose the model organism-based protein–protein interaction sub-network. In order to obtain high-quality PINs, we considered the interactions with the highest confidence limits (0.9) from String. Here, we used this platform to explore the relation between different SRGs and generated an interrelation network. We also used this platform to study PINs between SRGs and bacterial secretion systems.

### 4.5. Statistical Analysis

All of the genes coding for stress-responsive enzymes and stress regulators were selected based on the BLAST score (Table S3). To access the distribution of SRGs within different phylotypes of Rs, the data obtained from BLAST results were arranged in a raw data matrix, and a heatmap was generated using Morpheus (https://software.broadinstitute.org/morpheus/) (accessed on 25 May 2022). The cluster analysis was performed with the help of Past 3.17 using binary matrix data (based on the presence or absence of an ortholog) [80]. The neighbor-joining method was used to construct a cluster dendrogram using 1000 as the bootstrap value.

## 5. Conclusions

This study provides a detailed report of the SRGs of Rs, their distribution, classification, and interaction with bacterial secretion systems. This is the first report of SRGs across Rs genomes. From this investigation, it is proposed that the SRGs of DdSR play a pivotal role among all other clusters. Multivariate analysis with SRGs provides evidence in support of a purifying selection of the stress management system in Rs. Interactions between SRGs and bacterial secretion systems in Rs were also elucidated for the first time.

## Figures and Tables

**Table 1:** Reference dataset for bacterial stress-responsive enzymes and factors.

Sl. No.	Regulator	Stress Response	Reported Candidates	References
1	OxyR	Oxidative stress	*Salmonella enterica*	[10]
2	DksA	Oxidative stress	-	[11]
3	NsrR	NO	*S. enterica*	[12]
4	FNR	Oxygen limitation	*E. coli*	[13]
5	DNR	Nitrosative stress	-	[14]
6	Bae	Membrane stress	*-*	[15]
7	Cpx	Misfolded OMPs at acid pH	*S. enterica*	[16]
8	Rcs	Membrane stress	*-*	[15]
9	σ^E^	Membrane stress	*S. enterica*	[17]
10	RseA	Membrane stress	-	[18]
11	DegS	Membrane stress	-	[18]
12	RecA	DNA damage	*-*	[19]
13	LexA	DNA damage	*Vibrio cholerae*	[20]
14	FruR	Carbohydrate starvation	*E. coli*	[21]
15	SpoT	Amino acid starvation	*S. enterica*	[22]
16	FUR	Iron starvation	*S. enterica*	[23]
17	ZUR	Zinc starvation	*S. enterica*	[24]
18	SrrA/SrrB	Oxygen limitation	*S. enterica*	[25]
19	ResD/ResE	Oxygen limitation	*S. enterica*	[26]
20	ArcB/ArcA	Oxygen limitation	*E. coli*	[27]
21	DosR/DosS	Oxygen limitation	*M. tuberculosis*	[28]
22	Psp	Envelop stress	*S. enterica*	[29]
23	Rex	Oxygen limitation	*S. aureus*	[30]
24	NarQ/NarP	Oxygen limitation	*S. enterica*	[31]
25	NarX/NarL	Oxygen limitation	*E. coli*	[32]
26	Trg	Oxygen limitation	*-*	[33]
27	Tsr	Oxygen limitation	*E. coli*	[34]
28	Aer	Oxygen limitation	*E. coli*	[34]
29	H-NS	Temperature	*S. flexneri*	[35]
30	Hha	Temperature	*E. coli*	[36]
31	GmaR	Temperature	*L. monocytogenes*	[37]
32	MogR	Temperature	*L. monocytogenes*	[37]
33	BvgA/BvgS	Temperature	*B. pertussis*	[38]
34	RovA	Temperature	*Y. pestis*	[39]
35	SlyA	Temperature	*S. enterica*	[40]
36	PhoP/PhoQ	Temperature	*Y*. *enterocolitica*	[41]
37	PmrA/PmrB	Temperature	*Y*. *enterocolitica*	[41]
38	OmpR	Envelope	*S. enterica*	[42]
39	Dps	Nutrient limitation	*R. solanacearum*	[7]
40	ArsA	Acid pH	*H. pylori*	[43]
41	BvgA	Temperature regulates	*B. pertussis*	[38]
42	CsrA	Starvation	*S. flexneri*	[44]
43	DosR (NosR)	Hypoxia/NO^.^	*M. tuberculosis*	[45]
44	DtxR	Iron deprivation	*C. diphtheriae*	[46]
45	MntR	Manganese starvation	*Bacillus subtilis*	[47]
46	GmaR	Temperature	*Listeria monocytogenes*	[37]
47	σS	Starvation	*S. enterica*	[18]

**Table 2:** Functional classification of stress-responsive genes (SRG) of Rs.

Sl. No.	SRG	KO No.	Function	Cluster
1	nlpD	K06194	Lipoprotein NlpD | (GenBank) putative outer membrane metallopeptidase lipoprotein nlpD/	**MSR**
2	degQ	K04772	Serine protease DegQ (EC:3.4.21.-) | (GenBank) putative serine protease do-like precursor (degP)	
3	rseB	K03598	Sigma-E factor negative regulatory protein RseB | (GenBank) rseB; Sigma-E factor regulatory protein	
4	rseA	K03597	Sigma-E factor negative regulatory protein RseA | (GenBank) rseA; Sigma-E factor negative regulatory protein	
5	rpoE	K03088	RNA polymerase sigma-70 factor, ECF subfamily (misfolded OMPs at acid pH; required for virulence) | (GenBank) rpoE	
6	lexA	K01356	Repressor LexA (EC:3.4.21.88) (Regulates toxin production) | (GenBank) lexA	
7	recX	K03565	Regulatory protein (Modulates recA activity) | (GenBank) recX	
8	recA	K03553	Catalyzes the hydrolysis of ATP in the presence of single-stranded DNA, RecA | (GenBank) recAn	
9	baeR	K07664	Two-component system, OmpR family, response regulator BaeR for mdtABCD and acrD | (GenBank) baeR	**OSR**
10	baeS	K07642	Two-component system, OmpR family, sensor histidine kinase BaeS (EC:2.7.13.3) | (GenBank) baeS	
11	ompR	K07659	Two-component system, OmpR family, phosphate regulon response regulator OmpR | (GenBank) ompR	
12	ompR	K07638	Two-component system, OmpR family (EC:2.7.13.3) | (GenBank) putative sensory histidine kinase in Two-component regulatory system with OmpR	
13	envZ	K07638	Two-component system, OmpR family, osmolarity sensor histidine kinase EnvZ (EC:2.7.13.3) | (GenBank) envZ	
14	rcsB	K07687	Two-component system, NarL family, captular synthesis response regulator RcsB | (GenBank) putative response regulator receiver	
15	Kata	K03781	Catalase (EC:1.11.1.6) | (GenBank) kata	**OxSR**
16	katB	K03781	Catalase [EC:1.11.1.6] (Paraquat-inducible catalase isozyme B) | (GenBank) katB	
17	katG	K03782	Catalase-peroxidase (EC:1.11.1.21) | (GenBank) katG	
18	oxyR	K04761	LysR family transcriptional regulator, hydrogen peroxide-inducible genes activator | (GenBank) oxyR	
19	sodB	K04564	Superoxide dismutase, Fe-Mn family (EC:1.15.1.1) | (GenBank) sodB	
20	sodC	K04565	Superoxide dismutase, Cu-Zn family (EC:1.15.1.1) | (GenBank) sodC	
21	Dps	K04047	Starvation-inducible DNA-binding protein | (GenBank) putative DNA-binding protein	
22	nsrR	K13771	Rrf2 family transcriptional regulator, nitric oxide-sensitive transcriptional repressor (regulates genes required for virulence) | (GenBank) nsrR	**NxSR**
23	norB	K04561	Nitric oxide reductase subunit B (transmembrane protein) (EC:1.7.2.5) | (GenBank) norB	
24	aniA	K00368	Nitrite reductase (NO-forming) (EC:1.7.2.1) | (GenBank) ainA	
25	Fnr	K01420	CRP/FNR family transcriptional regulator, anaerobic regulatory protein | (GenBank) fnr	**DdSR**
26	Dnr	K21563	CRP/FNR family transcriptional regulator, dissimilatory nitrate respiration regulator | (GenBank) putative transcription regulator protein	
27	dskA	K06204	DnaK suppressor protein | (GenBank) dskA	
28	rpoZ	K03060	DNA-directed RNA polymerase subunit omega (EC:2.7.7.6) | (GenBank) rpoZ	
29	rpoS	K03087	RNA polymerase nonessential primary-like sigma factor (regulates expression of plasmid virulence genes) | (GenBank) rpoS	
30	rpoB	K03043	DNA-directed RNA polymerase subunit beta (EC:2.7.7.6) | (GenBank) RNA polymerase, beta subunit	
31	rpoA	K03040	DNA-directed RNA polymerase subunit alpha (EC:2.7.7.6) | (GenBank) RNA polymerase, alpha subunit	
32	polA	K02335	DNA polymerase I (EC:2.7.7.7) | (GenBank) polA	
33	h-ns	K03746	DNA-binding protein H-NS | (GenBank) Histone-like nucleoid-structuring protein H-NS	
34	Fur	K03711	Fur family transcriptional regulator, ferric uptake regulator | (GenBank) fur	
35	Gmk	K00942	Guanylate kinase | (GenBank) gmk	

## Data Availability

The genomes of *Ralstonia solanacearum* were downloaded from the NCBI RefSeq database (https://www.ncbi.nlm.nih.gov/genome/genomes/490) (accessed on 31 March 2022).

## Supplementary Materials

The following are available online at https://www.mdpi.com/article/10.3390/pathogens11070730/s1. Figure S1: Cluster dendrogram based on the distribution of stress-responsive genes within *R. solanacerium* species complex; Table S1: Secondary dataset for stress-responsive enzymes and factors of *R. solanacearum* using KO orthologs; Table S2: Secondary dataset for stress-responsive enzymes and factors of *R. solanacearum* using KO orthologs derived from STRING results; Table S3: Normalized (10%) pBLAST 2.2.26 score for the SRGs of *R. solanacearum*; Table S4: Bacterial secretion systems of Rs.
