# Supplementary material for: Interrelation between Stress Management and Secretion Systems of Ralstonia solanacearum: An In Silico Assessment
Source: Pathogens. 2022 Jun 27;11(7):730. doi: 10.3390/pathogens11070730 (PMC9325324; doi:10.3390/pathogens11070730)
Supplement: Supplementary file 1 [file pathogens-11-00730-s001.zip › pathogens-1710911-supplementary.pdf]

**Table S1.** Secondary data set for stress responsive enzymes and factors of *R. solanacearum* using KO orthologs.

| Sl. No. | Enzyme/Name | Coded by | KO No. | Quarry Sequence from *Ralstonia solanacearum* strain GMI1000 |
|---|---|---|---|---|
| 1 | Catalase | katE | K03781 | MSFPSRSLRVSLLALAALTAFHAGAVTLTRDNGAPVGDNQNSQTAGPNGPVLLQDV<br>QLIQKLQRFDRERIPERVVHARGTGAHGVFVASKDISDLTRAKVFTPGEETPVFVRFS<br>SVIHSGHSPETLRDPRGFATKFYTREGNWDLVGNNLPVFFIRDAMKFPDMVHSLKPS<br>PDSNVQDPNRFFDFFSHQPESTHMLTRVYTDYGTPTSYREMDGNSVHAYKFVNAKG<br>EVHYVKFHWKSLQGQKGLSAKEVEAIQGKDFNHMTRDLIANIDKGNYPKWDLYIQ<br>VLKPEQLAKFDFDPLDATKVWPDVPEVKVGTMTLNKNPENVFQETEQAALAPSNLV<br>PGIEPSEDRLLQGRIFSYADTQLYRVGTNGLQLPVNRPRTSAANYNQDGDLNAGARK<br>GSVNYQPSAHADLSDAATVKYSQLPVSGTTQQQRIARTQNFKQAGDYYRSLTAKEQ<br>ANLVSNLAGDLGQVKDDGVKYTMLSYFQKADAGYGRALAQAVKADPARVESLAA<br>KLQD |
| 2 | Catalase | katG | K03782 | MGPEPEAAGIEEQGLGWSSRFGTGKGTDAITSGLEVTWTTTPTQWSNNFFQNLFGYE<br>WELTKSPAGAHQWVAKDAAETIPDAHDPSAKHLPTMLTTDLSLRFDPAYEKISRRFY<br>EHPEQFADAFARAWFKLTHRDMGPRARYLGPEVPAEALIWQDPIPAVDHKLIDTQDI<br>AALKARILASGLSVSQLVSTAWASASTFRGSDMRGGANGARIRLAPQKDWEANQPA<br>QLAKVLETLESIQGAFNGAQSGGKKVSLADLIVLAGCAGVEQAAKNAGHAVEVPFT<br>PGRMDAAQAQTDVESFAVLEPIADGFRNYQKGKYTLPAEALLVDKAQLLTLTAPEM<br>TVLVGGLRVLDTNIGQTRHGVFTQRPESLTNDFFVNLLDMGTEWKATDGRDVFEGR<br>DRATGALKWTGTRVDLVFGSHSQLRALAEVYGSADAQAKFVRDFVAAWDKVMNL<br>DRFDLA |
| 3 | Nitric-oxide reductase | norB | K04561 | MNTNHRLWRWLGLIFVLSFGALGYLGRQIYLAAPPIPQAVVTTAGDVLFTGEQIQRG<br>QQAWLAAGGQQLGTVWGHGSYVAPDWSADWLHREATALQAIHTQQQFGSAASLT<br>AAQQAAVDVTVKEEMRRNTYDAARDSITVSKERARAIHGVAQHFEALFGTDPSLAT<br>LRDQYAMATGALPDQADREALAAFFFWTSWAAATDRPGETDVSYTSNWPHEPLVG<br>NTMTSDAAMWSMVSIILLLAGIAAMLWLHGSGKHEEDAKPLPADPFLNVVATPSMK<br>ATRKYFFAVIGLILVQIGMGAITAHYAVEGEAFFGIPLAQVLPYVISRTVHTQLGVLW<br>IATAWLATGLYIAPLLSGREPKFQKLGVDLLFWALIFIVVGSIATGWLGTLQHRGADF<br>GFWIGNQGLEYTSMGRVWQLLLFVGLLFWVALLGRALWPALKTPSESRGLIAMVFL<br>SATCIGGFYSTSLVWGQHTHYSMIEYWRWWLVHLWVEGFFEVFATAVVALLFTRL<br>GLVRAASANRAIVAETIVFLFGGILGTLHHLYFTGTPTSIISVGAVFSALEVVPLTLIGL<br>EALQTYRRMRGTPWLGAYKWPVLCFVAVGFWNTVGAGLLGFAINPPASLYYVQGL<br>NMTAAHGHAALFGVYGMLGIGLMLFCLRGLYARSLHADRLLKPAYWGLNIGLAM<br>MVFMSLLPAGIYQAWASITKGMWYARSPEVVHSKLMETLVWLRVPGDIVFAGGAFI<br>LALYALRLLRRPPSRQAATAPATATR |
| 4 | Superoxide dismutase | sodB | K04564 | MAHTLPPLPYALDALAPHISKETLEFHYGKHHQTYVTNLNNLIPGTEFENLSLEDIVK<br>KSSGGLFNNAAQVWNHTFYWNGLKPNGGGAPAGALADAINAKWGSFDKFKEEFTK<br>VAIGTFGSGWAWLVKKADGSLDLVSTSNAATPLTTDAKPLLTCDVWEHAYYIDYRN<br>ARPKYVEAFWSLVNWDFVASNFA |
| 5 | Superoxide dismutase | sodC | K04565 | MKQLVIGLAAIGLMAGCASNAGTGASTGAPAAGTSALATLAPKSGSNVQGTIKLLQ<br>QGDSRVAMAVDIAGLPPNGMFGFHVHEKGDCSSPDGMSAGGHFNPTGQPHGDPRS<br>GPHHAGDIPMLQSDASGKAVGSIVLNGVTLTPGATSLVGRAVIVHAGMDDYKTQPT<br>GNSGGRVACGVIVAN |
| 6 | Hydrogen peroxide-inducible genes activator | oxyR | K04761 | MIAMTLTELKYIVAVARERHFGRAAEACFVSQPTLSVAIKKLEDELAVQIFERGASEV<br>SVTPVGEQIVTQAQHVLEQTMAIREIAKQGMDPLAGPLRLGVIYTIGPYLLPALVKQ<br>MIDTVPQMPLMLQENFTARLVELLKQGEIDCAIMAEPFPEAGLMTVPLYDEPFVVAV<br>PRGHALAGSSAVDPEALKQQTMLLLGNGHCFRDHVLNVCPELSRFSQNADGIQKTF<br>EGSSLETIRHMVASGVGITVLPRTSVPDMQPAADLLAYVPFQEPVPDRRVVLAWRKS<br>FTRVAAIEAVAKAVAQCSLPGIKPLPVTPLVH |
| 7 | dnak suppressor protein | dksA | K06204 | MSSKKLLTEAEILKMGDKDYMNEAQLAFFKDRLEKLRDDILKNAGQTTEHLRETVI<br>VPDPADRATIEEEHALELRTRDRERKLLKKVEQSIARIDEGNYGYCDETGEPIGIPRLL<br>ARPTATLTLEAQERREKRQKLFGD |
| 8 | nitric oxide-sensitive transcriptional repressor | nsrR | K13771 | MRLTDYTDYSLRTLIYVAVHPDVLVTIQQIADAFGIPKNHLIKIVQGLGQNGFLHTVR<br>GRAGGITLGRPAVEINIGDVVRATEPDFSLVECFHVNDNHCIITRVCGLRGVLAAALQ<br>AYFEVLDTYTLQDLIERPAALNRVLAEGVAVPMPQSGKGRTPKAAPAAGSRTRKSG |
| 9 | dissimilatory nitrate respiration regulator | DNR | K21563 | MHETRDIGDLLKHSPLFEGLDAEDLATLARDSHQIRLARHDFVFHRGDCADGFYVV<br>AVGTIKLVLPGAHGHNKVIEFFGPGEYFGEPFMFLNQPHAADAQALEDSLLIRIGKPTI<br>DAALDIHPTLARRMLTGLSSRLHTLMCDIETVNLQSASQRLIGYLLMLPRKLDRARFP<br>CSKTLVASKLGLAPATLSRVLQQLIRDGLILVEGRDVVIHSAAALQRQLRAG |
| 10 | oxidative stress resistance two-component response regulator | ompR | K07659 | MENSGHKILVVDDDPRLRDLLRRYLGEQGFTVLVAENATAMNKLWLRERFDLLVL<br>DLMMPGEDGLSICRRLRGANDQTPIIMLTAKGEDVDRIVGLEMGADDYLPKPFNPRE<br>LIARIHAVLRRRGPAEIPGAPSETPETFAFGDFVLNLATRTLKKNDEEITLTTGEFSVLK<br>VFARHPRQPLSREKLMEMARGREYEVFDRSLDVQISRLRKLIEPDASNPRFIQTVWGL<br>GYVFIPDGAK |
| 11 | two-component response regulator | baeR | K07664 | MILIVEDEPKLAALMADYLRAAHYETEWVDDGTRAVQRVRETSPELVLLDLMLPGK<br>DGLEICREVRAFSDLPIVMVTARVEEIDRLLGLELGADDYICKPFSPRELVARIKTILR<br>RVQRSGAQSDSLLQIDNTAYSATLRGQRLDLTPVEFRLLSALANAPGRILSRANLLEQ<br>IYDDHRVVTDRTVDTHVKNLRRKMELVSPGTELIHSVYGVGYRLDA |
| 12 | Histidine kinase | baeS | K07642 | MRLSFGITSKLFVALFALSMVVALAMGAAVRWRFDTNFLDYVNAREAERMAVFAQ<br>SIESVYAEHGNWGVLRNDRQAWYRLIRREIGRTHAGPFGGPRGDAEPVPPFAPPPGD<br>VPPPGLPPPPPDAGDRPPQHPPGEGPGMHPPIWLLDAGRVPIVGDGPPPGPGSQWHAL<br>HHNGQTVGWLATPQRMHVPEGADQAFLAQQLRATWIIAALSVLLAAGVAILLARG |

| | | | | |
|---|---|---|---|---|
| | | | | VLAPMRRLAHATRRMAEGDYAARVDVHSGDEIGHLADDFNRLAETLEANQKMRR<br>QLTADISHELRTPLAVLRGELEALEDGVRALTPASLTSLQAEVGTLSKLIDDLYELSL<br>ADVGALAFHMESIDLADTVCSAAQGFTPRLMDKRIALQLDLPTGPSRDAAIQGDPQR<br>LRQLMQNLLENTLRYTDAGGTLRIHVHRAGAWWCLDVQDSAPGVPEAMLPRVFDR<br>LFRADPSRGRAQGGAGLGLSLCQTIVQTHGGTIDARPSPLGGLWIAIRLPAANSSVAD<br>HDPDR |
| 13 | periplasmic protein | cpxP/ spy | K06006 | MFAVTPRRSLAAVAAGLLLSCAVAYAQSSAPEASPRPPMAPHGMPHMPGGPGFMGP<br>HHGGLFLHGLKLTEAQRDKVFAIEYAQMPELREQRKAIEHARRDLREMVVSGQYDE<br>ARSRALTDALGRAVARETQLRAQADAKILQVLTPEQRKQISDREAQRVAAMEEEAG<br>PPPQPMM |
| 14 | captular synthesis response regulator | rcsB | K07687 | MQAPVRFAIADDHPGVVAAVRHLVCRVEGFEVVGEATSADGLLALLGRVHCDVVIT<br>DYAMPASRHGDGIVLLEFLLRRHPGVRVVVLTMLETHAVVDRMLRAGIRVIASKAD<br>SPHHILEGVHAALAGRAYLSPRFGPRLDSAHPGHAPDDHGALDRLGKRELEVLRMY<br>VGGETVSEIARRLNRSVKTISAQKQKSMRKLRLATEAALFDFAAQHGLLGTTGR |
| 15 | histidine kinase | rcsC | K07677 | MPQRLLTKTGRYSRRVLYGCTAALSIAIVGAVLLLGRGIIDQYGDRQVARFVRTSGE<br>VRTEVDGLSARLAQFADLYEGVWNLRQNDVVPTRRYAGKLAADRGATVTGADLT<br>ATPVTLISSLDRPADGARLAMALRVLRDVSGAPLMDAQKLGVTLDGFLYTPDATFL<br>AAFPALRPADLRSAREQGPQPFIRERIAAVEQRIASSAPEAVRGRRPLWYPADETNGH<br>ASVSQLIVPMYRDNARVLTIALTLPDAQFTRFFLRKESRRPGFFLLSQTGQRSLGDLP<br>ADLEERRLLDTVLANADWSRQAGAEPTTFYKDGVFFVSRRIAGPDWIAVYAYRWQ<br>DVWSDLQHEFFAGMVFCVLVLGLLWATAAYFDLRVTRPLLSDAKKLIEAEHFSKAII<br>DTLPIGIGVYSPETDSILLENSVAVRMLGNVSNEQRLRFYRYVMHERDAEPVPGARH<br>SFIEVRWERAEGQSSYIGVASSWTRFGGRSAVLLGLVDMNERKANEILLMEAKQTA<br>DEANRAKSMFLAIVGHEIRTPLHGAMGHLELLARSALSLEQREWVDMTSRSFDALLT<br>LVNDLLDSTKLEANALQISPVPMCPNEVLERCARSFGAAIVQGGVAFHCITDPDLDL<br>VVDGDDQRLTQILQNLLSNAAKFTERGTITIASRCLKREDGKVWARFEVADTGIGIPA<br>AMQSTIFNPLAQADDSISRRFGGTGLGLFLCRNLAHLMGGRISVRSEPGIGSAFRVDL<br>PFAQNTRAPAPPPPVLTGLSVELLCPVSQWRGMLAERLRRWG<br>ATVRPAEASAAEPLHIRLAVEADGRAWHAADLGTVPLLGTVFVTAKGPLHAHRDGQ<br>KIEVSSFTREGLLAALTELTGAAARPAETPVSMAVDEGLGADLDILVAEDDAVNRTLI<br>RHQLAALGCQRVRVAVDGVEALEMWLERRADLVITDLGMPRLDGIGLLHKLRELD<br>PRARVIATSASVSAEIKADVEQFSSLLQKPVSLGDLRRILRQEMLSRTASPTPAVAEPV<br>RADALDALLRQAFRTEWNKERKSIEQALAGKDMDALRRRVHRLQGALMALGADA<br>LVEELRGLQDIYSRADWALIAIRCHQLLAHVDASVG |
| 16 | RNA polymerase sigma-70 factor | rpoE (σE) | K03088 | MTIAPEQLDALRPHLLRFAQLQLRDPALAEDVVADTVLAVLEHPERFAGNASLKTY<br>VIGILKHKIIDAIRSGRREVRVSLLAGGASDETDQRTDEAVFDALFAADGHYLTPPSD<br>WGNPDAALSRREFFDILQMCVDRLPPRVGRVFMMREWLELETDEICQELEITATNA<br>WALLYRARMRLRACLDLHWFGNQGAAA |
| 17 | sigma-E factor negative regulatory protein | rseA | K03597 | MEFWGREMGQAQMQAADAGFAERISALMDGEVAAHEAAAAVELAKDGEGAGHW<br>REYQLIGDALRSEDLLGAHSTEAFLGRFSAKLDAEPHLLVPAVAQRAQTEERHRFLV<br>RPSWVRRVVPSTAVAAAVAAVSWVVVPQLRGPTGSGDASPALVAKAQQGAGAQA<br>VTVSAAENTPMIRDARLDEYLSAHRQSATNGVVVPYLRAVANGAASTQDSSQE |
| 18 | serine protease | degQ (degS) | K04772 | MLRRFWLFFAQAVTVVLAVWFVIATLKPEWLQRGKVAVQSGSPIVALKEVAPLGHS<br>GTTSNSYAEAAKVAMPAVVNIFSSKNAPKRNNPQANADPWFRFFFGDRLPEQRQEP<br>TASLGSGVIVSPEGYILTNHHVVDGADEIEVALTDGRKANAKVVGTDPETDLAVLKI<br>SLTNLPAITLGRLENVRVGDVVLAIGNPFGVGQTVTMGIVSALGRSHLGINTFENFIQ<br>TDAAINPGNSGGALVDAEGNLLGINTAIYSRSGGSLGIGFAIPVSLAKQVMESIISTGS<br>VVRGWIGVEPQDVTPEIAESFGLSRKDGALIAAVVQGGPADRAGLRPGDILINVNGES<br>IQDTTALLNSIAQLKPSTEAKVTVSRKGKPVELTIMVGKRPPPVRRNVPMPSPEDEQP<br>Q |
| 19 | recombinatio n protein | recA | K03553 | MEDGKKAASMSAEKQKALAAALAQIEKQFGKGSIMKMGDAEVEPVQVVSTGSLGL<br>DVALGVGGLPRGRVVEIYGPESSGKTTLTLQVVAEMQKLGGTCAFIDAEHALDVTY<br>ADKLGVKVPDLLISQPDTGEQALEIADALVRSGSVDLIVIDSVAALVPKAEIEGEMGD<br>ALPGLQARLMSQALRKLTGTIKRTNCLVIFINQIRMKIGVMFGSPETTTGGNALKFYA<br>SVRLDIRRIGSIKKGDEVVGNETKVKVVKNKVAPPFREAIFDILYGAGVSREGEIIDLG<br>VEAKVVEKSGAWYSYNGERIGQGRDNCREFLRENAELAREIENKVREHLGVTPMGA<br>VTLAEEVEED |
| 20 | recA repressor | lexA | K01356 | MATLTTRQQQIYDLIHQTIQRTGFPPTRAEIAAEFGFSSPNAAEEHLRALARKGVIELT<br>PGASRGIRLRAEGGASPHQFSLPSMGLMQLTLPLVGRVAAGSPILAAEHIDRQYQVD<br>PSLFSSRPDFLLKVRGMSMRDAGILDGDLLAVQRAAEAANGKIVVARLGDDVTVKR<br>FQRKGRQVELIAENPDFEPIHVDLDRDEFQLEGLAVGLIRPAAP |
| 21 | rna polymerase sigma s (sigma-38) factor | rpoS (σS) | K03087 | MPRQKAATSANPADETIGAVDGPNAPNGRSGRRARGAAPAASDDVEVVDPLIDEAP<br>AAEPADIADTSDANDSADESDEEDEDGEEAAPEEDFRTVLQAELAADTVQHYLNRIS<br>IKPLLTPAEELHFSTLAKAGEFAARQVMIERNLRLVVSIAKGYLNRGVPLLDLIEEGN<br>LGLMHAIEKFDPERGFRFSTYATWWIRQSIERAIMNQARTVRLPVHVIRELNQVLRA<br>KRHLEKSGIDGRDASIEDIAHLLGKTTEEVQDVLSLNEHTTSLDTPFDLDPGTSLLDFL<br>SDEHGASPDQEVAHRELSQLMKSWLARLSDKHRYVVERRFGLNHIEPATLEELAAE<br>MGLTRERVRQIQQEALVKLKRHFASQGVRKDAVL |
| 22 | fructose operon transcriptiona l repressor | fruR | K03435 | MTVRIKDVAAAAGVSVATVSRALAGGPVSEALRKRVDAAVAASGYRPNLSARRLR<br>SQQAQTVGLIVSDIRNPFFTSVSRAVEDAAYAAGLRVILCNSDEDPDKEAMYLRLME<br>EERVTGVILSPTRRIAESPAPVVLGFPVVLIDRAGPACGVDAVVLDNQQAAQALAEH<br>LAQHGYRRIGGLFGRTSSTGAERHAGVAAGLARHGLAIDARFILPTAEAAEAETLAW<br>LASGERPDALVASNAQSLLGVLRAIRRLSLDVPGDIAVAGFDNEPWTELAGPGVTVI<br>EQPVYDIGRLAMTMLQERLAQPELPTRRVLLTGRLVARGSTPQRRM |
| 23 | GTP diphosphokin ase | spot | K01139 | MFGPTSQPAAPPRQQVVSIAGLTEKLSYLKPADIKLVKEAFHFSDEAHLGQYRQSGEP<br>YITHPVAVAEICAGWKLDVQSIMAALLHDVIEDQGVTKSELAEKFGPKVAELVDGLT<br>KLDKLEFQSREQAQAESFRKMLLAMARDVRVILVKLADRTHNMRTLDHVPPEKRRR<br>IAGETMEIYAPIAHRLGLNTTYRELQELSFRIGSPFRYATLEKAVKAARGNRREVVSRI |

| | | | | |
|---|---|---|---|---|
| | | | | LEAAQRALGDAGVSAELTGREKTLYSIYRKMHDKQLSFSQVLDVYGFRVVVETQM<br>HCYMAVGALHGLYKPMPGKFKDYIAIPKINGYQSVHTTLVGPFGTPVEFQIRTREMN<br>QIAEAGVAAHWMYKQHHDEPDRAQQQAHQWLQSLLDIQSQSGDSQEFLEHVKIDL<br>FPDAVYVFTPKGEIRALPRGATALDFAYAVHSDLGNQCVAVKINNALLPLRTELKNG<br>DIVEVVTAPYSKPNPAWLTFVRTGKARAAIRHFLKTAKLDEAIQLGERLLEQALRQL<br>GIDMKAVPAQVWERIVQWTGNKAREDIFADLALGRRVAAVVARRIEIGLQEGGHEG<br>DEALIAAVHAFAGEEAPAVTVSGDEGMAMVFSPCCRPIPGDPIVGYIGKGEGLQIHVE<br>ECRVAKRLHGKDPEHWIDVMWAEHTTRAFDVSIKVLVRNTKGILARVAADLTSAD<br>ANVAHVSMEQEGGGQEATYMTFLIQVHDRVHLADVMRALRRNLDVIRIARDRGGE |
| 24 | ferric uptake regulator | fur (ZUR) | K03711 | MNSPAPRDVPTTSHPDAPEAAARARLRGLGSRVTEPRVRVLAALMQGDAPLSHQAV<br>CDALPADAGIDRVTVYRVLDWLVAGGIVHKTAGADRVFRFSLAEHDAAREAAHRA<br>HSHFHCTQCGRDFCLETATPPAVPTPALPAGFAADHAELTIKGCCADCAARHQTGNA<br>PCPN |
| 25 | dna protection during starvation | Dps | K04047 | MAKKNGGAAAAAQINIGIAEKDRKKIAEGLSRLLADTYSLYLKTHNFHWNVTGPMF<br>NTLHLMFETQYNELWTAVDSVAERIRALGYPAPGSYSEFARLSSIPEAKGVPAAEDM<br>IRELVAGHESVTRTARSLFPDVDKAADEPTADLLTQRMDIHEKTAWMLRSLLA |
| 26 | issimilatory nitrate respiration regulator | Fnr | K21563 | MHETRDIGDLLKHSPLFEGLDAEDLATLARDSHQIRLARHDFVFHRGDCADGFYVV<br>AVGTIKLVLPGAHGHNKVIEFFGPGEYFGEPFMFLNQPHAADAQALEDSLLIRIGKPTI<br>DAALDIHPTLARRMLTGLSSRLHTLMCDIETVNLQSASQRLIGYLLMLPRKLDRARFP<br>CSKTLVASKLGLAPATLSRVLQQLIRDGLILVEGRDVVIHSAAALQRQLRAG |
| 27 | anaerobic regulatory protein | Fnr | K01420 | MIPSTLKAQTSADVTRPTPLPQLKLVRDATQARQTVSPALSSQCANCMMHWVCIAG<br>AVPSTQQERVDRMVHTWRKVGKGEALFRAGDTFHALYAIRSGSFKTVVSHPNGSSH<br>VTGFQLAGETLGLDGIAADRHTCDAIALEDSTVCSMPFHCVEDLCQAIRPLQHRLHQ<br>LLAEEIVRESGLMLLLARLTAEARVATFLLNLSSRMQDRGYSGSCFTLRMTREEIGSY<br>LGMQLETVSRALSRFQREGWISVNGKQIELANKAALSEL |
| 28 | transcription regulator protein | H-NS | K03746 | MATYKDLLSQKAKLEEQLEAARQKELAEITAQVRQVVQEYGLTAEDIGLAPKRGGK<br>RAPKAAPVPKYRDPKTGATWTGRGRAPAWIGKNRDKYLIA |

**Table S2.** Secondary data set for stress responsive enzymes and factors of *R. solanacearum* using KO orthologs derived from STRING results

| **Sl. No.** | **Enzyme/Name** | **Coded by** | **KO No.** | **Quarry Sequence from *Ralstonia solanacearum* strain GMI1000** |
|---|---|---|---|---|
| 1 | lipoprotein | nlpD | K06194 | MNPPSPARAGRLAVAMVSAALLAACASSGNQAPVLDRTSRAGSAPAAPLEPPPPGY YRVKRGDTLYSIALRNGQAPRDLVTWNNIPNPNQIEVDQLIRVVPPNADASATGAVA TPVRPPNTTTQPIDAAPAATPPVVSSGASAGATDGAIALAWPAHGQVIGRFDDKANK GIDIGGKRGDAVTAADDGKVIHVGPLRGYGNLVIIKHNDTFLTAYGNNDKVLVTEQS TVKKGQKIAEMGSTDADRVKLHFEVRRNGKPVDPMRFLPPQ |
| 2 | serine protease | degQ | K04772 | MLRRFWLFFAQAVTVVLAVWFVIATLKPEWLQRGKVAVQSGSPIVALKEVAPLGHS GTTSNSYAEAAKVAMPAVVNIFSSKNAPKRNNPQANADPWFRFFFGDRLPEQRQEP TASLGSGVIVSPEGYILTNHHVVDGADEIEVALTDGRKANAKVVGTDPETDLAVLKI SLTNLPAITLGRLENVRVGDVVLAIGNPFGVGQTVTMGIVSALGRSHLGINTFENFIQ TDAAINPGNSGGALVDAEGNLLGINTAIYSRSGGSLGIGFAIPVSLAKQVMESIISTGS VVRGWIGVEPQDVTPEIAESFGLSRKDGALIAAVVQGGPADRAGLRPGDILINVNGES IQDTTALLNSIAQLKPSTEAKVTVSRKGKPVELTIMVGKRPPPVRRNVPMPSPEDEQP Q |
| 3 | sigma-E factor negative regulatory protein | rseB | K03598 | MRESMSKVWHPVAGAGARKLQAVRRSVFLLLCVSALSVAAQSQQPEPMPRKEAAS WLTKIHRAALKQNYVGTLTYQRGSGMHSTRIQHYTDLFNNEYERVEALDGKQREM LRQNDVVRNLIYEVKLVVTEKQENKDSFPALLATTNGDVLDQYDMRHLPAERVAG MDCEVFQLDPKDGFRYAYRIWAERSSGLLIRAQTIGEDGKVLEQVAFSQVEVGVPSE KQKILAALKSVTGWNQYEVVSQPTNLAEQGWAITSPIKGFQKIREVRRPLGDIAPAG KSSSGFEVQQVVFSDGLAGLSLFIEPVSEKRSRREGFISQGATHVMVRRIADFWLTVV GEVPFATIKQFGAAVDYKPVSANAASRPASTP |
| 4 | DNA repair and recombination protein | recX | K03565 | MALPRQPLSLKARALGYLSRREHSRAELRRKLAPHAESVDEVEALLDWLEGENWLS NTRFAESVVHRRASRYGTARLMQELKTHELGDDALGEVKAQLQASEMARAKAVW ERRFGRPPADMAERAKQVRFMVARGFSRAVVSRIIQGADALLDDGDEPV |
| 5 | osmolarity sensor histidine kinase | ompR | K07638 | MRIPRPAALRRLLISVFGSLFWRTFMLIALLIAISLGAWFQSFRIFEREPRAQQIAMQIV SVVKLTRAALLYSDPARRRFLLLDLVQNEGIKVYPREKEDEYRTPQANPYLTQLVQR EIRNRLGQDTVIATAVNDIPGVWVSFQIEGDDYWVAISPDRFEHVPGLQWLWWSIAA LVLSVLGAAFITSRVNQPLKRLADTARAISAGDDPKSLPEGGGTEVAQANHSFNQMV RDLKQLEADRAVMLAGISHDLRTPLTRLRLETEMSPVDDQTRELMVADIEQMDAIIG QFLDYARPSGEMLEAVDLTELVRDTAPVYSAHDDIDLTLKLAPEAIARCNRMETQRI LDNLIENARRYGKTHDTGRAEITVSTALQGNEVVLCVADRGAGVPADQLALLTRPF YRLESARSEAKGAGLGMSIVSRILQRSGGRLTLENRTPPETGLVVSACYARG |
| 6 | sensor histidine kinase transcription regulator protein | envZ | K07638 | MRIPRPAALRRLLISVFGSLFWRTFMLIALLIAISLGAWFQSFRIFEREPRAQQIAMQIV SVVKLTRAALLYSDPARRRFLLLDLVQNEGIKVYPREKEDEYRTPQANPYLTQLVQR EIRNRLGQDTVIATAVNDIPGVWVSFQIEGDDYWVAISPDRFEHVPGLQWLWWSIAA LVLSVLGAAFITSRVNQPLKRLADTARAISAGDDPKSLPEGGGTEVAQANHSFNQMV RDLKQLEADRAVMLAGISHDLRTPLTRLRLETEMSPVDDQTRELMVADIEQMDAIIG QFLDYARPSGEMLEAVDLTELVRDTAPVYSAHDDIDLTLKLAPEAIARCNRMETQRI LDNLIENARRYGKTHDTGRAEITVSTALQGNEVVLCVADRGAGVPADQLALLTRPF YRLESARSEAKGAGLGMSIVSRILQRSGGRLTLENRTPPETGLVVSACYARG |
| 7 | catalase hydroperoxidase hpII oxidoreductase protein | kata | K03781 | MSFPSRSLRVSLLALAALTAFHAGAVTLTRDNGAPVGDNQNSQTAGPNGPVLLQDV QLIQKLQRFDRERIPERVVHARGTGAHGVFVASKDISDLTRAKVFTPGEETPVFVRFS SVIHSGHSPETLRDPRGFATKFYTREGNWDLVGNNLPVFFIRDAMKFPDMVHSLKPS PDSNVQDPNRFFDFFSHQPESTHMLTRVYTDYGTPTSYREMDGNSVHAYKFVNAKG EVHYVKFHWKSLQGQKGLSAKEVEAIQGKDFNHMTRDLIANIDKGNYPKWDLYIQ VLKPEQLAKFDFDPLDATKVWPDVPEVKVGTMTLNKNPENVFQETEQAALAPSNLV PGIEPSEDRLLQGRIFSYADTQLYRVGTNGLQLPVNRPRTSAANYNQDGDLNAGARK GSVNYQPSAHADLSDAATVKYSQLPVSGTTQQQRIARTQNFKQAGDYYRSLTAKEQ ANLVSNLAGDLGQVKDDGVKYTMLSYFQKADAGYGRALAQAVKADPARVESLAA KLQD |

**Table S3.** Normalised (10%) pBLAST 2.2.26 score for the SRGs of *R. solanacearum.*

| | **FJAT15252** | **FJAT1458** | **FJAT1458** | **FJAT1463** | **FJAT1463** | **FJAT15249** | **FJAT15252** | **FJAT15249** | **T78** | **EP1** | **T60** | **FJAT454** | **FJAT448** | **FJAT448** | **SL3882** |
|---|---|---|---|---|---|---|---|---|---|---|---|---|---|---|---|
| **katE** | 0 | 0 | 0 | 0 | 0 | 0 | 0 | 0 | 0 | 0 | 0 | 0 | 0 | 0 | 0 |
| **katG** | 98.71 | 98.71 | 98.71 | 98.71 | 98.71 | 98.71 | 98.71 | 98.71 | 98.82 | 98.39 | 98.82 | 98.71 | 98.71 | 98.71 | 98.82 |
| **norB** | 0 | 0 | 0 | 0 | 0 | 0 | 0 | 0 | 0 | 0 | 0 | 0 | 0 | 0 | 0 |
| **sodB** | 81.73 | 81.73 | 81.73 | 81.73 | 81.73 | 81.73 | 81.73 | 81.73 | 81.73 | 81.73 | 81.73 | 81.73 | 81.73 | 81.73 | 81.73 |
| **sodC** | 86.63 | 86.63 | 86.63 | 86.63 | 86.63 | 86.63 | 86.63 | 86.63 | 86.63 | 87.19 | 86.63 | 86.63 | 86.63 | 86.63 | 86.63 |
| **oxyR** | 96.11 | 96.11 | 96.11 | 96.11 | 96.11 | 96.11 | 96.11 | 96.11 | 96.11 | 95.65 | 96.11 | 96.11 | 96.11 | 96.11 | 96.11 |
| **dksA** | 90.32 | 90.32 | 90.32 | 90.32 | 90.32 | 90.32 | 90.32 | 90.32 | 90.32 | 89.61 | 90.32 | 90.32 | 90.32 | 90.32 | 90.32 |
| **nsrR** | 100 | 100 | 100 | 100 | 100 | 100 | 100 | 100 | 100 | 100 | 100 | 100 | 100 | 100 | 100 |
| **dnr** | 0 | 0 | 0 | 0 | 0 | 0 | 0 | 0 | 0 | 0 | 0 | 0 | 0 | 0 | 0 |
| **ompR** | 92.18 | 92.18 | 92.18 | 92.18 | 92.18 | 92.18 | 92.18 | 92.18 | 92.18 | 92.18 | 92.18 | 92.18 | 92.18 | 92.18 | 92.18 |
| **baeR** | 35.31 | 35.31 | 35.31 | 35.31 | 35.31 | 35.31 | 35.31 | 35.31 | 35.31 | 35.08 | 35.31 | 35.31 | 35.31 | 35.31 | 35.31 |
| **baeS** | 0 | 0 | 0 | 0 | 0 | 0 | 0 | 0 | 0 | 0 | 0 | 0 | 0 | 0 | 0 |
| **gmk** | 100 | 100 | 100 | 100 | 100 | 100 | 100 | 100 | 99.34 | 99.34 | 99.34 | 99.34 | 99.34 | 99.34 | 99.34 |
| **rcsB** | 99.32 | 99.32 | 99.32 | 99.32 | 99.32 | 99.32 | 99.32 | 99.32 | 99.32 | 99.54 | 99.32 | 99.32 | 99.32 | 99.32 | 99.32 |
| **rcsC** | 0 | 0 | 0 | 0 | 0 | 0 | 0 | 0 | 0 | 0 | 0 | 0 | 0 | 0 | 0 |
| **rpoE** | 90.27 | 90.27 | 90.27 | 90.27 | 90.27 | 90.27 | 90.27 | 90.27 | 90.27 | 90.52 | 90.27 | 90.27 | 90.27 | 90.27 | 90.27 |
| **rseA** | 60.7 | 60.7 | 60.7 | 60.7 | 60.7 | 60.7 | 60.7 | 60.7 | 60.7 | 60.23 | 60.7 | 60.7 | 60.7 | 60.7 | 60.7 |
| **degS** | 94.42 | 94.42 | 94.42 | 94.42 | 94.42 | 94.42 | 94.42 | 94.42 | 94.42 | 94.42 | 94.42 | 94.42 | 94.42 | 94.42 | 94.42 |
| **recA** | 82.15 | 82.15 | 82.15 | 82.15 | 82.15 | 82.15 | 82.15 | 82.15 | 82.15 | 82.15 | 82.15 | 82.15 | 82.15 | 82.15 | 82.15 |
| **lexA** | 99.77 | 99.77 | 99.77 | 99.77 | 99.77 | 99.77 | 99.77 | 99.77 | 99.77 | 99.77 | 99.77 | 99.77 | 99.77 | 99.77 | 99.77 |
| **rpoS** | 82 | 82 | 82 | 82 | 82 | 82 | 82 | 82 | 82 | 82 | 82 | 82 | 82 | 82 | 82 |
| **fruR** | 85.03 | 85.03 | 85.03 | 85.03 | 85.03 | 85.03 | 85.03 | 85.03 | 85.03 | 85.03 | 85.03 | 85.03 | 85.03 | 85.03 | 85.03 |
| **spoT** | 98.48 | 98.48 | 98.48 | 98.48 | 98.48 | 98.48 | 98.48 | 98.48 | 98.35 | 98.48 | 98.48 | 98.48 | 98.48 | 98.48 | 98.48 |
| **fur** | 71.22 | 71.22 | 71.22 | 71.22 | 71.22 | 71.22 | 71.22 | 71.22 | 71.22 | 71.22 | 71.22 | 71.22 | 71.22 | 71.22 | 71.22 |
| **dps** | 88.92 | 88.92 | 88.92 | 88.92 | 88.92 | 88.92 | 88.92 | 88.92 | 89.21 | 88.92 | 89.21 | 88.92 | 88.92 | 88.92 | 89.21 |
| **fnr** | 0 | 0 | 0 | 0 | 0 | 0 | 0 | 0 | 0 | 0 | 0 | 0 | 0 | 0 | 0 |
| **rpoA** | 88.87 | 88.87 | 88.87 | 88.87 | 88.87 | 88.87 | 88.87 | 88.87 | 88.87 | 88.87 | 88.87 | 88.87 | 88.87 | 88.87 | 88.87 |
| **h-ns** | 96.34 | 96.34 | 96.34 | 96.34 | 96.34 | 96.34 | 96.34 | 96.34 | 92.15 | 96.34 | 96.34 | 96.34 | 96.34 | 96.34 | 96.34 |
| katA | 0 | 0 | 0 | 0 | 0 | 0 | 0 | 0 | 0 | 0 | 0 | 0 | 0 | 0 | 0 |
| envZ | 93.26 | 93.26 | 93.26 | 93.26 | 93.26 | 93.26 | 93.26 | 93.26 | 93.26 | 93.26 | 93.26 | 93.26 | 93.26 | 93.26 | 93.26 |
| ompR | 93.26 | 93.26 | 93.26 | 93.26 | 93.26 | 93.26 | 93.26 | 93.26 | 93.26 | 93.26 | 93.26 | 93.26 | 93.26 | 93.26 | 93.26 |
| nlpD | 78.34 | 78.34 | 78.34 | 78.34 | 78.34 | 78.34 | 78.34 | 78.34 | 78.34 | 78.34 | 78.34 | 78.34 | 78.34 | 78.34 | 78.34 |
| degQ | 94.42 | 94.42 | 94.42 | 94.42 | 94.42 | 94.42 | 94.42 | 94.42 | 94.42 | 94.42 | 94.42 | 94.42 | 94.42 | 94.42 | 94.42 |
| recX | 96.21 | 96.21 | 96.21 | 96.21 | 96.21 | 96.21 | 96.21 | 96.21 | 96.21 | 97.16 | 96.21 | 96.21 | 96.21 | 96.21 | 96.21 |
| rseB | 92.84 | 92.84 | 92.84 | 92.84 | 92.84 | 92.84 | 92.84 | 92.84 | 92.84 | 93.24 | 92.84 | 92.84 | 92.84 | 92.84 | 92.84 |

**Table S3.** (Cont.)

| | FJAT454 | SEPPX05 | MAFF 211471 | MAFF 211472 | RS24 | HA4-1 | FJAT91 | FJAT91 | FJAT91 | SL3300 | FJAT15353 | FJAT15353 | FJAT15353 | FJAT1303 | FJAT1303 |
|---|---|---|---|---|---|---|---|---|---|---|---|---|---|---|---|
| **katE** | 0 | 0 | 0 | 0 | 0 | 0 | 0 | 0 | 0 | 0 | 0 | 0 | 0 | 0 | 0 |
| **katG** | 98.71 | 98.28 | 98.82 | 98.82 | 98.39 | 98.82 | 98.82 | 98.82 | 98.82 | 98.82 | 98.39 | 98.39 | 98.39 | 98.39 | 98.39 |
| **norB** | 0 | 0 | 0 | 0 | 0 | 0 | 0 | 0 | 0 | 0 | 0 | 0 | 0 | 0 | 0 |
| **sodB** | 81.73 | 80.96 | 81.73 | 81.73 | 81.73 | 81.98 | 81.73 | 81.73 | 81.73 | 81.73 | 81.73 | 81.73 | 81.73 | 81.73 | 81.73 |
| **sodC** | 86.63 | 86.63 | 86.07 | 86.07 | 87.19 | 86.63 | 86.07 | 86.07 | 86.07 | 86.63 | 86.63 | 86.63 | 86.63 | 86.63 | 86.63 |
| **oxyR** | 96.11 | 96.11 | 96.11 | 96.11 | 95.65 | 96.11 | 96.11 | 96.11 | 96.11 | 96.11 | 96.11 | 96.11 | 96.11 | 96.11 | 96.11 |
| **dksA** | 90.32 | 90.32 | 90.32 | 90.32 | 90.32 | 90.32 | 90.32 | 90.32 | 90.32 | 90.32 | 90.32 | 90.32 | 90.32 | 90.32 | 90.32 |
| **nsrR** | 100 | 99.72 | 100 | 100 | 100 | 99.72 | 100 | 100 | 100 | 100 | 99.72 | 99.72 | 99.72 | 100 | 100 |
| **dnr** | 0 | 0 | 0 | 0 | 0 | 0 | 0 | 0 | 0 | 0 | 0 | 0 | 0 | 0 | 0 |
| **ompR** | 92.18 | 92.18 | 92.18 | 92.18 | 92.18 | 92.18 | 92.18 | 92.18 | 92.18 | 92.18 | 92.18 | 92.18 | 92.18 | 92.18 | 92.18 |
| **baeR** | 35.31 | 35.31 | 35.31 | 35.31 | 35.08 | 35.08 | 35.31 | 35.31 | 35.31 | 35.31 | 35.31 | 35.31 | 35.31 | 35.08 | 35.08 |
| **baeS** | 0 | 0 | 0 | 0 | 0 | 0 | 0 | 0 | 0 | 0 | 0 | 0 | 0 | 0 | 0 |
| **gmk** | 99.34 | 100 | 99.34 | 99.34 | 99.34 | 99.34 | 99.34 | 99.34 | 99.34 | 99.34 | 99.34 | 99.34 | 99.34 | 99.34 | 99.34 |
| **rcsB** | 99.32 | 98.63 | 99.77 | 99.77 | 98.86 | 99.77 | 99.77 | 99.77 | 99.77 | 99.32 | 99.77 | 99.77 | 99.77 | 99.77 | 99.77 |
| **rcsC** | 0 | 0 | 0 | 0 | 0 | 0 | 0 | 0 | 0 | 0 | 0 | 0 | 0 | 0 | 0 |
| **rpoE** | 90.27 | 90.27 | 90.27 | 90.27 | 90.52 | 90.52 | 90.27 | 90.27 | 90.27 | 90.27 | 89.03 | 89.03 | 89.03 | 89.03 | 89.03 |
| **rseA** | 60.7 | 60 | 60.7 | 60.7 | 60.23 | 60 | 60.7 | 60.7 | 60.7 | 60.7 | 60.23 | 60.23 | 60.23 | 60.23 | 60.23 |
| **degS** | 94.42 | 94.42 | 94.42 | 94.42 | 94.42 | 94.17 | 94.42 | 94.42 | 94.42 | 94.42 | 94.42 | 94.42 | 94.42 | 94.42 | 94.42 |
| **recA** | 82.15 | 82.15 | 82.15 | 82.15 | 82.15 | 82.15 | 82.15 | 82.15 | 82.15 | 82.15 | 82.15 | 82.15 | 82.15 | 82.15 | 82.15 |
| **lexA** | 99.77 | 100.7 | 99.77 | 99.77 | 99.77 | 99.77 | 99.77 | 99.77 | 99.77 | 99.77 | 99.77 | 99.77 | 99.77 | 99.77 | 99.77 |
| **rpoS** | 82 | 76.08 | 82 | 82 | 82 | 82 | 82 | 82 | 82 | 82 | 82 | 82 | 82 | 82 | 82 |
| **fruR** | 85.03 | 85.03 | 85.03 | 85.03 | 85.03 | 85.03 | 85.03 | 85.03 | 85.03 | 85.03 | 85.03 | 85.03 | 85.03 | 85.03 | 85.03 |
| **spoT** | 98.48 | 98.48 | 98.48 | 98.48 | 98.48 | 98.48 | 98.48 | 98.48 | 98.48 | 98.48 | 98.48 | 98.48 | 98.48 | 98.48 | 98.48 |
| **fur** | 71.22 | 71.22 | 71.22 | 71.22 | 71.22 | 71.22 | 71.22 | 71.22 | 71.22 | 71.22 | 71.22 | 71.22 | 71.22 | 71.22 | 71.22 |
| **dps** | 88.92 | 89.21 | 89.21 | 89.21 | 88.92 | 88.92 | 89.21 | 89.21 | 89.21 | 89.21 | 89.21 | 89.21 | 89.21 | 89.21 | 89.21 |
| **fnr** | 0 | 0 | 0 | 0 | 0 | 0 | 0 | 0 | 0 | 0 | 0 | 0 | 0 | 0 | 0 |
| **rpoA** | 88.87 | 88.87 | 88.87 | 88.87 | 88.87 | 88.87 | 88.87 | 88.87 | 88.87 | 88.87 | 88.87 | 88.87 | 88.87 | 88.87 | 88.87 |
| **h-ns** | 96.34 | 96.34 | 96.34 | 96.34 | 96.34 | 97.91 | 96.34 | 96.34 | 96.34 | 96.34 | 96.34 | 96.34 | 96.34 | 92.15 | 92.15 |
| **katA** | 0 | 0 | 0 | 0 | 0 | 0 | 0 | 0 | 0 | 0 | 0 | 0 | 0 | 0 | 0 |
| **envZ** | 93.26 | 93.04 | 93.26 | 93.26 | 93.26 | 92.93 | 92.93 | 92.93 | 92.93 | 92.93 | 92.93 | 92.93 | 92.93 | 92.93 | 92.93 |
| **ompR** | 93.26 | 93.04 | 93.26 | 93.26 | 93.26 | 92.93 | 93.26 | 93.26 | 93.26 | 93.26 | 93.26 | 93.26 | 93.26 | 93.26 | 93.26 |
| **nlpD** | 78.34 | 78.34 | 78.34 | 78.34 | 78.34 | 78.34 | 78.34 | 78.34 | 78.34 | 78.34 | 78.34 | 78.34 | 78.34 | 78.34 | 78.34 |
| **degQ** | 94.42 | 94.42 | 94.42 | 94.42 | 94.42 | 94.17 | 94.42 | 94.42 | 94.42 | 94.42 | 94.42 | 94.42 | 94.42 | 94.42 | 94.42 |
| **recX** | 96.21 | 97.16 | 96.21 | 96.21 | 97.16 | 96.21 | 96.21 | 96.21 | 96.21 | 96.21 | 97.16 | 97.16 | 97.16 | 97.16 | 97.16 |
| **rseB** | 92.84 | 93.37 | 92.84 | 92.84 | 93.24 | 90.19 | 92.84 | 92.84 | 92.84 | 92.84 | 93.24 | 93.24 | 93.24 | 93.24 | 93.24 |

**Table S3.** (Cont.)

| | YC40-M | SL3822 | RUN2279 | CQPS-1 | B2 | MAFF 301560 | MAFF 241647 | 204 | 203 | 202 | FJAT15244 | RSCM | FJAT15244 | FJAT15244 | SL3755 |
|---|---|---|---|---|---|---|---|---|---|---|---|---|---|---|---|
| **katE** | 0 | 0 | 0 | 0 | 0 | 0 | 0 | 0 | 0 | 0 | 0 | 0 | 0 | 0 | 0 |
| **katG** | 98.71 | 98.82 | 97.42 | 98.49 | 98.6 | 98.39 | 98.71 | 98.49 | 98.49 | 98.49 | 98.82 | 98.39 | 98.82 | 98.82 | 98.92 |
| **norB** | 0 | 0 | 0 | 0 | 0 | 0 | 0 | 0 | 0 | 0 | 0 | 0 | 0 | 0 | 0 |
| **sodB** | 81.73 | 81.73 | 81.98 | 81.73 | 81.73 | 81.73 | 81.73 | 81.73 | 81.73 | 81.73 | 81.73 | 81.73 | 81.73 | 81.73 | 81.73 |
| **sodC** | 86.63 | 86.63 | 86.07 | 87.19 | 85.52 | 87.19 | 86.63 | 87.19 | 87.19 | 87.19 | 86.07 | 87.19 | 86.07 | 86.07 | 86.63 |
| **oxyR** | 95.65 | 96.11 | 95.33 | 67.19 | 96.11 | 95.65 | 95.65 | 96.11 | 96.11 | 96.11 | 96.11 | 96.11 | 96.11 | 96.11 | 96.11 |
| **dksA** | 90.32 | 90.32 | 90.32 | 90.32 | 90.32 | 90.32 | 90.32 | 90.32 | 90.32 | 90.32 | 90.32 | 90.32 | 90.32 | 90.32 | 90.32 |
| **nsrR** | 99.72 | 100 | 97.44 | 100 | 100 | 100 | 99.72 | 100 | 100 | 100 | 100 | 100 | 100 | 100 | 99.72 |
| **dnr** | 0 | 0 | 10.46 | 0 | 10.71 | 0 | 0 | 0 | 0 | 0 | 0 | 0 | 0 | 0 | 0 |
| **ompR** | 92.18 | 92.18 | 91.77 | 92.18 | 92.18 | 92.18 | 92.18 | 92.18 | 92.18 | 92.18 | 92.18 | 92.18 | 92.18 | 92.18 | 92.18 |
| **baeR** | 35.08 | 35.31 | 35.08 | 35.08 | 35.31 | 35.08 | 35.08 | 35.08 | 35.08 | 35.08 | 35.08 | 35.31 | 35.08 | 35.08 | 35.08 |
| **baeS** | 0 | 0 | 0 | 0 | 0 | 0 | 0 | 0 | 0 | 0 | 0 | 0 | 0 | 0 | 0 |
| **gmk** | 99.34 | 99.34 | 100 | 100 | 99.34 | 99.34 | 99.34 | 100 | 100 | 100 | 99.34 | 100 | 99.34 | 99.34 | 100 |
| **rcsB** | 99.77 | 99.32 | 96.13 | 98.18 | 99.32 | 98.86 | 99.77 | 97.49 | 97.49 | 97.49 | 99.77 | 99.77 | 99.77 | 99.77 | 99.77 |
| **rcsC** | 0 | 0 | 0 | 0 | 0 | 0 | 0 | 0 | 0 | 0 | 0 | 0 | 0 | 0 | 0 |
| **rpoE** | 90.27 | 90.27 | 87.78 | 88.53 | 89.03 | 90.52 | 90.27 | 88.53 | 88.53 | 88.53 | 89.03 | 90.52 | 89.03 | 89.03 | 89.03 |
| **rseA** | 60.7 | 60.7 | 58.84 | 60.23 | 60 | 60.23 | 60.7 | 60.23 | 60.23 | 60.23 | 60.23 | 60.23 | 60.23 | 60.23 | 60.23 |
| **degS** | 94.42 | 94.42 | 93.8 | 94.42 | 94.42 | 94.42 | 94.42 | 94.42 | 94.42 | 94.42 | 94.42 | 94.42 | 94.42 | 94.42 | 94.42 |
| **recA** | 82.15 | 82.15 | 81.02 | 82.15 | 82.15 | 82.15 | 82.15 | 82.15 | 82.15 | 82.15 | 82.15 | 82.15 | 82.15 | 82.15 | 82.15 |
| **lexA** | 99.77 | 99.77 | 97.91 | 99.77 | 99.77 | 99.77 | 99.77 | 99.77 | 99.77 | 99.77 | 99.77 | 0 | 99.77 | 99.77 | 99.77 |
| **rpoS** | 82 | 82 | 80.55 | 82 | 82 | 82 | 82 | 82 | 82 | 82 | 82 | 23.92 | 82 | 82 | 82 |
| **fruR** | 85.03 | 85.03 | 84.88 | 85.03 | 85.03 | 85.03 | 85.03 | 85.03 | 85.03 | 85.03 | 85.03 | 85.03 | 85.03 | 85.03 | 85.03 |
| **spoT** | 98.48 | 98.48 | 98.15 | 98.48 | 98.41 | 98.48 | 98.48 | 98.48 | 98.48 | 98.48 | 98.48 | 98.48 | 98.48 | 98.48 | 98.48 |
| **fur** | 70.93 | 71.22 | 69.48 | 70.64 | 71.22 | 71.22 | 70.93 | 70.64 | 70.64 | 70.64 | 71.22 | 71.22 | 71.22 | 71.22 | 71.22 |
| **dps** | 88.92 | 89.21 | 89.21 | 89.21 | 88.92 | 88.92 | 88.92 | 89.21 | 89.21 | 89.21 | 89.21 | 89.21 | 89.21 | 89.21 | 88.92 |
| **fnr** | 0 | 0 | 10.46 | 0 | 10.71 | 0 | 0 | 0 | 0 | 0 | 0 | 0 | 0 | 0 | 0 |
| **rpoA** | 88.87 | 88.87 | 88.87 | 88.87 | 88.87 | 88.87 | 88.87 | 88.87 | 88.87 | 88.87 | 88.87 | 88.87 | 88.87 | 88.87 | 88.87 |
| **h-ns** | 96.34 | 96.34 | 96.34 | 90.05 | 92.15 | 96.34 | 96.34 | 95.81 | 95.81 | 95.81 | 96.34 | 91.1 | 96.34 | 96.34 | 96.34 |
| **katA** | 0 | 0 | 0 | 0 | 0 | 0 | 0 | 0 | 0 | 0 | 0 | 0 | 0 | 0 | 0 |
| **envZ** | 92.93 | 92.93 | 93.04 | 93.04 | 92.93 | 93.26 | 93.26 | 93.26 | 93.26 | 93.26 | 93.26 | 93.26 | 93.26 | 93.26 | 92.93 |
| **ompR** | 93.26 | 93.26 | 93.04 | 93.26 | 92.93 | 93.26 | 93.26 | 93.26 | 93.26 | 93.26 | 93.26 | 93.26 | 93.26 | 93.26 | 92.93 |
| **nlpD** | 78.34 | 78.34 | 78.7 | 78.34 | 78.34 | 78.34 | 78.34 | 78.34 | 78.34 | 78.34 | 78.34 | 0 | 78.34 | 78.34 | 78.34 |
| **degQ** | 94.42 | 94.42 | 93.8 | 94.42 | 94.42 | 94.42 | 94.42 | 94.42 | 94.42 | 94.42 | 94.42 | 94.42 | 94.42 | 94.42 | 94.42 |
| **recX** | 97.16 | 96.21 | 95.9 | 97.16 | 95.58 | 97.16 | 97.16 | 97.16 | 97.16 | 97.16 | 97.16 | 97.16 | 97.16 | 97.16 | 97.16 |
| **rseB** | 92.84 | 92.84 | 93.77 | 93.24 | 90.19 | 93.24 | 92.84 | 93.24 | 93.24 | 93.24 | 93.24 | 93.1 | 93.24 | 93.24 | 92.97 |

**Table S3.** (Cont.)

| | YQ | MAFF 241648 | MAFF 211479 | MAFF 211491 | UW763 | FJ1003 | T117 | 362200 | T42 | SL3730 | SL2729 | RUN2474 | FJAT442 | FJAT442 | FJAT1452 |
|---|---|---|---|---|---|---|---|---|---|---|---|---|---|---|---|
| **katE** | 0 | 0 | 0 | 0 | 0 | 0 | 0 | 0 | 0 | 0 | 0 | 0 | 0 | 0 | 0 |
| **katG** | 98.82 | 98.28 | 98.28 | 98.82 | 98.71 | 98.39 | 98.82 | 98.82 | 98.82 | 98.82 | 98.82 | 97.42 | 98.71 | 98.71 | 98.71 |
| **norB** | 0 | 0 | 0 | 0 | 0 | 0 | 79.56 | 0 | 0 | 0 | 0 | 0 | 0 | 0 | 0 |
| **sodB** | 81.73 | 81.98 | 81.98 | 81.73 | 81.73 | 81.73 | 81.73 | 81.98 | 81.73 | 81.73 | 81.73 | 80.96 | 81.73 | 81.73 | 81.73 |
| **sodC** | 86.07 | 87.19 | 87.19 | 86.07 | 86.63 | 87.19 | 86.63 | 86.63 | 86.63 | 86.63 | 86.63 | 86.07 | 86.63 | 86.63 | 86.63 |
| **oxyR** | 96.11 | 96.11 | 96.11 | 96.11 | 96.11 | 96.11 | 96.11 | 96.11 | 96.11 | 96.11 | 96.11 | 95.33 | 95.65 | 95.65 | 95.65 |
| **dksA** | 90.32 | 90.32 | 90.32 | 90.32 | 90.32 | 90.32 | 90.32 | 90.32 | 90.32 | 90.32 | 90.32 | 90.32 | 90.32 | 90.32 | 90.32 |
| **nsrR** | 100 | 100 | 100 | 100 | 99.72 | 100 | 100 | 99.72 | 100 | 100 | 100 | 97.44 | 99.72 | 99.72 | 99.72 |
| **dnr** | 0 | 0 | 0 | 0 | 0 | 0 | 11.57 | 0 | 0 | 0 | 0 | 10.46 | 0 | 0 | 0 |
| **ompR** | 92.18 | 92.18 | 92.18 | 92.18 | 92.18 | 92.18 | 92.18 | 92.18 | 92.18 | 92.18 | 92.18 | 91.77 | 92.18 | 92.18 | 92.18 |
| **baeR** | 35.31 | 35.31 | 35.31 | 35.31 | 35.76 | 35.08 | 35.31 | 35.08 | 35.31 | 35.31 | 35.31 | 35.08 | 35.08 | 35.08 | 35.08 |
| **baeS** | 0 | 0 | 0 | 0 | 0 | 0 | 0 | 0 | 0 | 0 | 0 | 0 | 0 | 0 | 0 |
| **gmk** | 99.34 | 100 | 100 | 100 | 100 | 99.34 | 99.34 | 99.34 | 99.34 | 99.34 | 99.34 | 100 | 99.34 | 99.34 | 99.34 |
| **rcsB** | 99.77 | 98.63 | 98.63 | 98.63 | 99.77 | 99.77 | 99.32 | 99.77 | 99.32 | 98.41 | 99.32 | 96.13 | 99.77 | 99.77 | 99.77 |
| **rcsC** | 0 | 0 | 0 | 0 | 0 | 0 | 0 | 0 | 0 | 0 | 0 | 0 | 0 | 0 | 0 |
| **rpoE** | 90.27 | 90.52 | 90.52 | 90.52 | 90.52 | 89.03 | 90.27 | 90.52 | 89.53 | 89.53 | 89.53 | 87.78 | 99.27 | 99.27 | 99.27 |
| **rseA** | 60.7 | 60.23 | 60.23 | 60.23 | 60.7 | 60.23 | 60.7 | 60 | 60.7 | 60.7 | 60.7 | 58.84 | 60.7 | 60.7 | 60.7 |
| **degS** | 94.42 | 94.42 | 94.42 | 94.42 | 94.42 | 94.42 | 71.34 | 94.17 | 94.42 | 94.42 | 94.42 | 93.8 | 94.42 | 94.42 | 94.42 |
| **recA** | 82.15 | 82.15 | 82.15 | 82.15 | 82.15 | 82.15 | 82.15 | 82.15 | 82.15 | 82.15 | 82.15 | 81.02 | 82.15 | 82.15 | 82.15 |
| **lexA** | 99.77 | 99.77 | 99.77 | 99.77 | 99.77 | 99.77 | 99.77 | 99.77 | 99.77 | 99.77 | 99.77 | 97.91 | 99.77 | 99.77 | 99.77 |
| **rpoS** | 82 | 82 | 82 | 82 | 82 | 82 | 82 | 82 | 81.6 | 82 | 82 | 80.55 | 80.55 | 80.55 | 80.55 |
| **fruR** | 85.03 | 85.03 | 85.03 | 85.03 | 85.03 | 85.03 | 85.03 | 85.03 | 85.03 | 85.03 | 85.03 | 84.88 | 85.03 | 85.03 | 85.03 |
| **spoT** | 98.48 | 98.48 | 98.48 | 98.48 | 98.48 | 98.48 | 98.48 | 98.48 | 98.48 | 98.48 | 98.48 | 97.09 | 98.48 | 98.48 | 98.48 |
| **fur** | 71.22 | 71.22 | 71.22 | 71.22 | 71.22 | 71.22 | 71.22 | 71.22 | 71.22 | 71.22 | 71.22 | 69.77 | 70.93 | 70.93 | 70.93 |
| **dps** | 89.21 | 89.21 | 88.92 | 88.92 | 89.21 | 89.21 | 89.21 | 88.92 | 89.21 | 89.21 | 89.21 | 89.21 | 88.92 | 88.92 | 88.92 |
| **fnr** | 0 | 0 | 0 | 0 | 0 | 0 | 0 | 0 | 0 | 0 | 0 | 10.46 | 0 | 0 | 0 |
| **rpoA** | 88.87 | 88.87 | 88.87 | 88.87 | 88.87 | 88.87 | 88.87 | 88.87 | 88.87 | 88.87 | 88.87 | 88.87 | 88.87 | 88.87 | 88.87 |
| **h-ns** | 96.34 | 98.95 | 98.95 | 98.95 | 100 | 96.34 | 96.34 | 97.91 | 96.34 | 96.34 | 96.34 | 90.58 | 96.34 | 96.34 | 96.34 |
| **katA** | 0 | 0 | 0 | 0 | 0 | 0 | 39.54 | 0 | 0 | 0 | 0 | 0 | 0 | 0 | 0 |
| **envZ** | 93.26 | 93.26 | 93.26 | 93.26 | 93.15 | 93.26 | 93.26 | 92.93 | 93.26 | 93.26 | 93.26 | 93.04 | 93.26 | 93.26 | 93.26 |
| **ompR** | 93.26 | 93.26 | 93.26 | 93.26 | 93.15 | 93.26 | 93.26 | 93.93 | 93.26 | 93.26 | 93.26 | 93.04 | 93.26 | 93.26 | 93.26 |
| **nlpD** | 78.34 | 78.34 | 78.34 | 78.34 | 78.34 | 78.34 | 78.34 | 78.34 | 78.34 | 78.34 | 78.34 | 78.7 | 78.34 | 78.34 | 78.34 |
| **degQ** | 94.42 | 94.42 | 94.42 | 94.42 | 94.42 | 94.42 | 94.42 | 94.17 | 94.17 | 94.17 | 94.17 | 93.8 | 94.42 | 94.42 | 94.42 |
| **recX** | 96.21 | 95.58 | 95.58 | 95.58 | 96.53 | 97.16 | 96.21 | 96.21 | 96.21 | 96.21 | 96.21 | 95.9 | 97.16 | 97.16 | 97.16 |
| **rseB** | 92.84 | 92.97 | 92.97 | 92.97 | 93.1 | 93.24 | 92.84 | 90.19 | 92.84 | 92.84 | 92.84 | 93.77 | 92.84 | 92.84 | 92.84 |

**Table S3.** (Cont.)

| | FJAT1452 | FJAT445 | FJAT 445 | SY1 | MAFF 311693 | SL2330 | FJAT1 5304 | FJAT15 340 | FJAT 1530 4 | FJAT15 340 | FJAT15 304 | FJAT1534 0 | UW386 | Rs-SY1 | FJAT1303 |
|---|---|---|---|---|---|---|---|---|---|---|---|---|---|---|---|
| **katE** | 0 | 0 | 0 | 0 | 0 | 0 | 0 | 0 | 0 | 0 | 0 | 0 | 0 | 0 | 0 |
| **katG** | 98.71 | 98.71 | 98.71 | 98.82 | 98.28 | 98.92 | 98.39 | 98.39 | 98.39 | 98.39 | 98.39 | 98.39 | 97.53 | 98.39 | 98.39 |
| **norB** | 0 | 0 | 0 | 0 | 0 | 0 | 0 | 0 | 0 | 0 | 0 | 0 | 0 | 0 | 0 |
| **sodB** | 81.73 | 81.73 | 81.73 | 81.73 | 81.98 | 81.73 | 81.73 | 81.73 | 81.73 | 81.73 | 81.73 | 81.73 | 81.73 | 81.73 | 81.73 |
| **sodC** | 86.63 | 86.63 | 86.63 | 86.63 | 87.19 | 86.63 | 86.63 | 86.63 | 86.63 | 86.63 | 86.63 | 86.63 | 86.07 | 86.63 | 86.63 |
| **oxyR** | 95.65 | 95.65 | 95.65 | 96.11 | 96.11 | 96.11 | 96.11 | 96.11 | 96.11 | 96.11 | 96.11 | 96.11 | 95.65 | 96.11 | 96.11 |
| **dksA** | 90.32 | 90.32 | 90.32 | 90.32 | 90.32 | 90.32 | 90.32 | 90.32 | 90.32 | 90.32 | 90.32 | 90.32 | 90.32 | 90.32 | 90.32 |
| **nsrR** | 99.72 | 99.72 | 99.72 | 99.72 | 100 | 99.72 | 100 | 100 | 100 | 100 | 100 | 100 | 97.72 | 100 | 100 |
| **dnr** | 0 | 0 | 0 | 0 | 0 | 0 | 0 | 0 | 0 | 0 | 0 | 0 | 0 | 0 | 0 |
| **ompR** | 92.18 | 92.18 | 92.18 | 92.39 | 92.18 | 92.18 | 92.18 | 92.18 | 92.18 | 92.18 | 92.18 | 92.18 | 92.18 | 92.18 | 92.18 |
| **baeR** | 35.08 | 35.08 | 35.08 | 35.08 | 35.31 | 35.08 | 35.08 | 35.08 | 35.08 | 35.08 | 35.08 | 35.08 | 32.57 | 35.08 | 35.08 |
| **baeS** | 0 | 0 | 0 | 0 | 0 | 0 | 0 | 0 | 0 | 0 | 0 | 0 | 0 | 0 | 0 |
| **gmk** | 99.34 | 99.34 | 99.34 | 100 | 100 | 100 | 99.34 | 99.34 | 99.34 | 99.34 | 99.34 | 99.34 | 99.56 | 99.34 | 99.34 |
| **rcsB** | 99.77 | 99.77 | 99.77 | 99.77 | 98.63 | 99.77 | 99.77 | 99.77 | 99.77 | 99.77 | 99.77 | 99.77 | 97.04 | 99.77 | 99.77 |
| **rcsC** | 0 | 0 | 0 | 0 | 0 | 0 | 0 | 0 | 0 | 0 | 0 | 0 | 0 | 0 | 0 |
| **rpoE** | 99.27 | 99.27 | 99.27 | 90.52 | 90.52 | 89.03 | 89.03 | 89.03 | 89.03 | 89.03 | 89.03 | 89.03 | 88.53 | 89.03 | 89.03 |
| **rseA** | 60.7 | 60.7 | 60.7 | 60 | 60.23 | 60.23 | 60.23 | 60.23 | 60.23 | 60.23 | 60.23 | 60.23 | 59.77 | 60.23 | 60.23 |
| **degS** | 94.42 | 94.42 | 94.42 | 94.17 | 94.42 | 94.42 | 94.42 | 94.42 | 94.42 | 94.42 | 94.42 | 94.42 | 94.42 | 94.42 | 94.42 |
| **recA** | 82.15 | 82.15 | 82.15 | 82.15 | 82.15 | 82.15 | 82.15 | 82.15 | 82.15 | 82.15 | 82.15 | 82.15 | 82.15 | 82.15 | 82.15 |
| **lexA** | 99.77 | 99.77 | 99.77 | 99.77 | 99.77 | 99.77 | 99.77 | 99.77 | 99.77 | 99.77 | 99.77 | 99.77 | 95.59 | 99.77 | 99.77 |
| **rpoS** | 80.55 | 80.55 | 80.55 | 81.47 | 82 | 82 | 82 | 82 | 82 | 82 | 82 | 82 | 81.08 | 82 | 82 |
| **fruR** | 85.03 | 85.03 | 85.03 | 85.03 | 85.03 | 85.03 | 85.03 | 85.03 | 85.03 | 85.03 | 85.03 | 85.03 | 85.19 | 85.03 | 85.03 |
| **spoT** | 98.48 | 98.48 | 98.48 | 98.48 | 98.48 | 98.48 | 98.48 | 98.48 | 98.48 | 98.48 | 98.48 | 98.48 | 97.09 | 98.48 | 98.48 |
| **fur** | 70.93 | 70.93 | 70.93 | 70.64 | 71.22 | 71.22 | 71.22 | 71.22 | 71.22 | 71.22 | 71.22 | 71.22 | 68.19 | 71.22 | 71.22 |
| **dps** | 88.92 | 88.92 | 88.92 | 89.21 | 89.21 | 89.21 | 89.21 | 89.21 | 89.21 | 89.21 | 89.21 | 89.21 | 88.63 | 89.21 | 89.21 |
| **fnr** | 0 | 0 | 0 | 0 | 0 | 0 | 0 | 0 | 0 | 0 | 0 | 0 | 0 | 0 | 0 |
| **rpoA** | 88.87 | 88.87 | 88.87 | 88.87 | 88.87 | 88.87 | 88.87 | 88.87 | 88.87 | 88.87 | 88.87 | 88.87 | 88.87 | 88.87 | 88.87 |
| **h-ns** | 96.34 | 96.34 | 96.34 | 96.34 | 98.43 | 96.34 | 92.15 | 92.15 | 92.15 | 92.15 | 92.15 | 92.15 | 96.34 | 96.34 | 92.15 |
| katA | 0 | 0 | 0 | 0 | 0 | 0 | 0 | 0 | 0 | 0 | 0 | 0 | 0 | 0 | 0 |
| envZ | 93.26 | 93.26 | 93.26 | 92.93 | 93.26 | 92.93 | 93.26 | 93.26 | 93.26 | 93.26 | 93.26 | 93.26 | 92.83 | 93.26 | 93.26 |
| ompR | 93.26 | 93.26 | 93.26 | 92.93 | 93.26 | 92.93 | 93.26 | 93.26 | 93.26 | 93.26 | 93.26 | 93.26 | 92.83 | 93.26 | 93.26 |
| nlpD | 78.34 | 78.34 | 78.34 | 78.34 | 78.34 | 78.34 | 78.34 | 78.34 | 78.34 | 78.34 | 78.34 | 78.34 | 78.34 | 78.34 | 78.34 |
| degQ | 94.42 | 94.42 | 94.42 | 94.17 | 94.42 | 94.42 | 94.42 | 94.42 | 94.42 | 94.42 | 94.42 | 94.42 | 94.42 | 94.42 | 94.42 |
| recX | 97.16 | 97.16 | 97.16 | 96.21 | 95.58 | 97.16 | 97.16 | 97.16 | 97.16 | 97.16 | 97.16 | 97.16 | 96.85 | 97.16 | 97.16 |
| rseB | 92.84 | 92.84 | 92.84 | 90.19 | 92.97 | 92.97 | 93.24 | 93.24 | 93.24 | 93.24 | 93.24 | 93.24 | 92.84 | 93.24 | 93.24 |

**Table S3.** (Cont.)

| | MAFF 211491 | Rs5 | T95 | SL2064 | KACC 10722 | SL3175 | T98 | T11 | SL23 12 | T82 | T101 | T12 | T51 | UW163 | SL3103 |
|---|---|---|---|---|---|---|---|---|---|---|---|---|---|---|---|
| **katE** | 0 | 0 | 39.41 | 39.41 | 39.41 | 39.41 | 39.41 | 39.41 | 39.51 | 39.51 | 39.51 | 39.51 | 39.41 | 0 | 0 |
| **katG** | 98.28 | 90 | 91.29 | 91.29 | 91.29 | 91.61 | 91.61 | 91.29 | 91.51 | 91.51 | 91.51 | 91.51 | 91.29 | 89.46 | 98.82 |
| **norB** | 0 | 0 | 79.56 | 79.56 | 79.56 | 79.37 | 79.37 | 0 | 79.83 | 79.83 | 79.83 | 79.83 | 0 | 0 | 0 |
| **sodB** | 81.98 | 82.99 | 80.2 | 80.2 | 80.2 | 80.2 | 80.2 | 81.73 | 80.2 | 80.2 | 80.2 | 80.2 | 80.2 | 82.99 | 81.73 |
| **sodC** | 87.19 | 82.45 | 84.68 | 84.68 | 84.68 | 84.96 | 84.96 | 84.68 | 84.96 | 84.96 | 84.96 | 84.96 | 84.68 | 81.34 | 86.63 |
| **oxyR** | 96.11 | 92.22 | 94.56 | 94.56 | 94.56 | 94.56 | 94.56 | 96.11 | 94.56 | 94.56 | 94.56 | 94.56 | 94.56 | 93.62 | 96.11 |
| **dksA** | 90.32 | 88.17 | 84.59 | 84.59 | 84.59 | 84.59 | 84.95 | 84.59 | 84.59 | 84.59 | 84.59 | 84.59 | 84.59 | 88.17 | 90.32 |
| **nsrR** | 100 | 90.03 | 91.17 | 91.17 | 91.17 | 86.04 | 86.04 | 100 | 91.17 | 91.17 | 91.17 | 91.17 | 91.17 | 92.69 | 100 |
| **dnr** | 0 | 0 | 11.57 | 11.57 | 11.57 | 11.66 | 11.66 | 0 | 11.57 | 11.57 | 11.57 | 11.57 | 11.57 | 0 | 0 |
| **ompR** | 92.18 | 92.18 | 92.18 | 92.18 | 92.18 | 92.18 | 92.18 | 92.18 | 92.18 | 92.18 | 92.18 | 92.39 | 92.18 | 92.39 | 92.18 |
| **baeR** | 35.31 | 35.54 | 35.31 | 35.31 | 35.31 | 35.31 | 35.31 | 35.31 | 35.31 | 35.31 | 35.31 | 35.31 | 35.31 | 35.54 | 35.31 |
| **baeS** | 0 | 0 | 0 | 0 | 0 | 0 | 0 | 0 | 0 | 0 | 0 | 0 | 0 | 0 | 0 |
| **gmk** | 100 | 99.56 | 98.25 | 98.25 | 98.25 | 98.25 | 98.25 | 94.09 | 98.25 | 98.25 | 98.25 | 98.25 | 98.25 | 99.78 | 99.34 |
| **rcsB** | 98.63 | 81.32 | 89.29 | 89.29 | 89.29 | 88.84 | 88.84 | 68.11 | 89.75 | 89.75 | 89.75 | 89.75 | 89.29 | 81.32 | 99.77 |
| **rcsC** | 0 | 0 | 0 | 0 | 0 | 0 | 0 | 0 | 0 | 0 | 0 | 0 | 0 | 0 | 0 |
| **rpoE** | 90.52 | 84.04 | 85.04 | 85.04 | 85.04 | 85.04 | 85.04 | 90.27 | 85.04 | 85.04 | 85.04 | 85.04 | 85.04 | 84.29 | 90.27 |
| **rseA** | 60.23 | 57.91 | 56.51 | 56.51 | 56.51 | 56.05 | 56.05 | 27.67 | 56.51 | 56.51 | 56.51 | 56.51 | 56.51 | 57.91 | 60.7 |
| **degS** | 94.42 | 91.44 | 91.19 | 91.19 | 91.19 | 90.94 | 90.94 | 94.42 | 90.94 | 63.77 | 63.77 | 63.77 | 91.19 | 91.69 | 94.42 |
| **recA** | 82.15 | 81.16 | 78.61 | 78.61 | 78.61 | 78.47 | 78.47 | 82.15 | 78.61 | 78.61 | 78.61 | 78.61 | 78.61 | 78.61 | 82.15 |
| **lexA** | 99.77 | 96.52 | 93.97 | 93.97 | 93.97 | 93.97 | 93.97 | 51.74 | 93.97 | 93.97 | 93.97 | 93.97 | 93.97 | 96.29 | 99.77 |
| **rpoS** | 82 | 80.29 | 81.21 | 81.21 | 81.21 | 81.21 | 81.21 | 81.21 | 81.21 | 81.21 | 81.21 | 81.21 | 81.21 | 79.76 | 82 |
| **fruR** | 85.03 | 85.95 | 84.41 | 84.41 | 84.41 | 84.1 | 84.1 | 85.03 | 84.41 | 84.41 | 84.41 | 84.41 | 84.41 | 83.8 | 85.03 |
| **spoT** | 98.48 | 95.24 | 95.38 | 95.38 | 95.38 | 96.3 | 96.3 | 95.38 | 95.24 | 95.24 | 95.24 | 95.24 | 95.38 | 95.9 | 98.41 |
| **fur** | 71.22 | 66.86 | 68.6 | 68.6 | 68.6 | 69.19 | 69.19 | 68.6 | 68.6 | 68.6 | 68.6 | 68.6 | 68.6 | 68.02 | 71.22 |
| **dps** | 89.21 | 89.21 | 89.21 | 89.21 | 89.21 | 89.21 | 89.21 | 89.21 | 89.21 | 89.21 | 89.21 | 89.21 | 89.21 | 89.21 | 88.63 |
| **fnr** | 0 | 0 | 11.57 | 11.57 | 11.57 | 11.66 | 11.66 | 11.57 | 11.57 | 11.57 | 11.57 | 11.57 | 11.57 | 0 | 0 |
| **rpoA** | 88.87 | 88.72 | 88.57 | 88.57 | 88.57 | 88.57 | 88.57 | 88.87 | 88.57 | 88.57 | 88.57 | 88.57 | 88.57 | 88.57 | 88.87 |
| **h-ns** | 98.95 | 90.58 | 96.34 | 96.34 | 96.34 | 87.43 | 87.43 | 67.02 | 72.77 | 72.77 | 72.77 | 72.77 | 13.93 | 91.1 | 13.51 |
| **katA** | 0 | 0 | 39.54 | 39.54 | 39.54 | 39.54 | 39.54 | 0 | 39.64 | 39.64 | 39.64 | 39.64 | 39.54 | 0 | 0 |
| **envZ** | 93.26 | 90.54 | 91.09 | 91.09 | 91.09 | 90.76 | 90.76 | 93.26 | 83.04 | 91.09 | 91.09 | 83.04 | 91.09 | 89.89 | 93.26 |
| **ompR** | 93.26 | 90.54 | 91.09 | 91.09 | 91.09 | 90.76 | 90.76 | 93.26 | 83.04 | 91.09 | 91.09 | 83.04 | 91.09 | 89.89 | 93.26 |
| **nlpD** | 78.34 | 74.91 | 75.09 | 75.09 | 75.09 | 75.09 | 75.09 | 75.09 | 75.09 | 75.09 | 75.09 | 75.09 | 75.09 | 75.09 | 78.34 |
| **degQ** | 94.42 | 91.44 | 91.19 | 91.19 | 91.19 | 90.94 | 90.94 | 73.82 | 90.94 | 63.77 | 63.77 | 63.77 | 91.19 | 91.69 | 94.42 |
| **recX** | 95.58 | 91.8 | 94.01 | 94.01 | 94.01 | 94.95 | 94.95 | 94.01 | 94.64 | 94.64 | 94.64 | 94.64 | 94.01 | 92.43 | 97.16 |
| **rseB** | 92.97 | 90.98 | 92.57 | 92.57 | 92.57 | 92.57 | 92.57 | 92.57 | 92.31 | 92.31 | 92.31 | 92.31 | 92.57 | 91.91 | 92.84 |

**Table S3.** (Cont.)

| | CIAT_078 | IBSBF 2571 | KACC107 09 | IBSBF15 03 | UY031 | RS 488 | RS 489 | GMI10 00 | FJAT-1458 | FJAT-91 | PSI07 | OE1-1 | Po82 | RS10 | YC45 |
|---|---|---|---|---|---|---|---|---|---|---|---|---|---|---|---|
| **katE** | 0 | 0 | 0 | 0 | 0 | 0 | 0 | 0 | 0 | 0 | 39.41 | 0 | 0 | 0 | 0 |
| **katG** | 89.46 | 89.46 | 98.82 | 89.46 | 89.57 | 89.57 | 89.14 | 99.89 | 98.71 | 98.82 | 91.94 | 98.39 | 89.46 | 98.49 | 98.28 |
| **norB** | 0 | 0 | 0 | 0 | 0 | 0 | 0 | 0 | 0 | 0 | 79.37 | 0 | 0 | 0 | 0 |
| **sodB** | 82.99 | 82.23 | 81.73 | 82.99 | 82.99 | 82.99 | 82.99 | 81.73 | 81.73 | 81.73 | 80.2 | 81.73 | 82.99 | 81.98 | 81.98 |
| **sodC** | 81.34 | 81.34 | 86.63 | 81.34 | 83.57 | 83.57 | 84.12 | 87.74 | 86.63 | 86.07 | 84.96 | 87.19 | 81.34 | 87.19 | 87.19 |
| **oxyR** | 93.62 | 93.62 | 96.11 | 93.62 | 93 | 93 | 92.22 | 96.11 | 96.11 | 96.11 | 94.56 | 95.65 | 93.62 | 96.11 | 96.11 |
| **dksA** | 87.81 | 88.17 | 90.32 | 88.17 | 88.17 | 88.17 | 88.17 | 90.32 | 90.32 | 90.32 | 84.95 | 90.32 | 88.17 | 90.32 | 90.32 |
| **nsrR** | 92.59 | 92.59 | 99.43 | 92.59 | 91.17 | 91.17 | 82.34 | 100 | 100 | 100 | 86.04 | 100 | 92.59 | 100 | 100 |
| **dnr** | 0 | 0 | 0 | 0 | 0 | 0 | 0 | 0 | 0 | 0 | 11.66 | 0 | 0 | 0 | 0 |
| **ompR** | 92.39 | 92.39 | 92.18 | 92.39 | 92.39 | 92.39 | 92.18 | 92.18 | 92.18 | 92.18 | 92.18 | 92.18 | 92.39 | 91.36 | 92.18 |
| **baeR** | 35.54 | 35.76 | 35.31 | 35.54 | 35.31 | 35.31 | 35.08 | 35.31 | 35.31 | 35.31 | 35.31 | 35.08 | 35.54 | 35.31 | 32.12 |
| **baeS** | 0 | 0 | 0 | 0 | 0 | 0 | 0 | 0 | 0 | 0 | 0 | 0 | 0 | 0 | 0 |
| **gmk** | 99.78 | 99.34 | 99.34 | 98.78 | 99.34 | 99.34 | 99.56 | 100 | 100 | 99.34 | 98.03 | 99.34 | 99.78 | 100 | 100 |
| **rcsB** | 81.55 | 81.32 | 99.77 | 81.32 | 82.69 | 82.69 | 80.18 | 99.77 | 99.32 | 99.77 | 88.84 | 98.86 | 81.32 | 98.41 | 27.56 |
| **rcsC** | 0 | 0 | 0 | 0 | 0 | 0 | 0 | 0 | 0 | 0 | 0 | 0 | 0 | 0 | 0 |
| **rpoE** | 84.29 | 84.29 | 90.27 | 84.29 | 84.54 | 84.54 | 83.29 | 90.52 | 90.27 | 90.27 | 85.04 | 90.52 | 84.29 | 90.52 | 90.52 |
| **rseA** | 57.91 | 57.91 | 60.7 | 57.91 | 57.67 | 57.67 | 57.67 | 60.7 | 60.7 | 60.7 | 56.05 | 60.23 | 57.91 | 60.23 | 60.23 |
| **degS** | 91.69 | 91.69 | 94.42 | 91.69 | 91.56 | 91.32 | 91.56 | 94.42 | 94.42 | 94.42 | 90.94 | 94.42 | 91.69 | 94.42 | 94.42 |
| **recA** | 78.61 | 51.98 | 82.15 | 78.61 | 81.16 | 81.16 | 80.74 | 82.15 | 82.15 | 82.15 | 78.47 | 82.15 | 78.61 | 78.61 | 82.15 |
| **lexA** | 96.29 | 96.29 | 99.77 | 96.29 | 96.29 | 96.29 | 96.52 | 100.46 | 99.77 | 99.77 | 93.97 | 99.77 | 96.29 | 99.77 | 99.77 |
| **rpoS** | 73.32 | 79.76 | 82 | 79.76 | 79.89 | 79.89 | 80.16 | 82 | 82 | 82 | 81.21 | 82 | 79.76 | 82 | 81.47 |
| **fruR** | 83.64 | 83.8 | 85.03 | 83.8 | 83.49 | 83.49 | 83.49 | 85.03 | 85.03 | 85.03 | 84.26 | 85.03 | 83.8 | 85.03 | 85.03 |
| **spoT** | 64.27 | 95.77 | 98.41 | 95.9 | 96.17 | 96.17 | 95.44 | 98.61 | 98.48 | 98.48 | 96.3 | 98.48 | 95.77 | 98.48 | 98.48 |
| **fur** | 68.02 | 68.02 | 71.22 | 68.02 | 67.73 | 67.73 | 67.15 | 71.22 | 71.22 | 71.22 | 69.19 | 71.22 | 68.02 | 71.22 | 71.22 |
| **dps** | 89.21 | 89.21 | 89.21 | 89.21 | 88.63 | 88.63 | 88.92 | 89.21 | 88.92 | 89.21 | 89.21 | 88.92 | 89.21 | 89.21 | 89.21 |
| **fnr** | 0 | 0 | 0 | 0 | 0 | 0 | 0 | 0 | 0 | 0 | 11.66 | 0 | 0 | 0 | 0 |
| **rpoA** | 88.57 | 88.57 | 88.87 | 88.57 | 88.72 | 88.72 | 88.72 | 88.87 | 88.87 | 88.87 | 88.57 | 88.87 | 88.57 | 88.87 | 88.57 |
| **h-ns** | 93.19 | 92.15 | 89.01 | 92.15 | 13.93 | 13.93 | 13.93 | 100 | 96.34 | 96.34 | 95.81 | 96.34 | 82.15 | 97.91 | 98.95 |
| **katA** | 0 | 0 | 0 | 0 | 0 | 0 | 0 | 0 | 0 | 0 | 39.54 | 0 | 0 | 0 | 0 |
| **envZ** | 89.89 | 89.89 | 93.26 | 89.89 | 90.54 | 90.54 | 90.22 | 93.15 | 93.26 | 93.26 | 90.54 | 93.26 | 89.89 | 93.15 | 93.26 |
| **ompR** | 89.89 | 89.89 | 93.26 | 89.89 | 90.54 | 90.54 | 90.22 | 93.15 | 93.26 | 93.26 | 90.54 | 93.26 | 89.89 | 93.15 | 93.26 |
| **nlpD** | 75.09 | 75.09 | 78.34 | 75.09 | 75.09 | 75.09 | 75.09 | 78.34 | 78.34 | 78.34 | 75.09 | 78.34 | 75.09 | 78.34 | 78.34 |
| **degQ** | 91.69 | 91.69 | 94.42 | 91.69 | 91.56 | 91.32 | 91.56 | 94.42 | 94.42 | 94.42 | 90.94 | 94.42 | 91.69 | 94.42 | 94.42 |
| **recX** | 92.43 | 93.38 | 97.16 | 91.48 | 93.06 | 92.74 | 90.85 | 96.53 | 97.16 | 97.16 | 94.95 | 97.16 | 92.43 | 97.16 | 95.58 |
| **rseB** | 91.91 | 91.91 | 92.84 | 91.91 | 91.64 | 91.64 | 90.85 | 93.37 | 93.24 | 93.24 | 92.57 | 93.24 | 91.91 | 93.37 | 92.97 |

**Table S3.** (Cont.)

| | T25 | T110 | SL3022 | CFBP 8695 | CFBP 8697 |
|---|---|---|---|---|---|
| **katE** | 0 | 0 | 39.51 | 0 | 0 |
| **katG** | 98.92 | 59.14 | 54.52 | 86.27 | 85.27 |
| **norB** | 0 | 0 | 79.63 | 0 | 0 |
| **sodB** | 81.73 | 80.46 | 80.2 | 82.99 | 81.98 |
| **sodC** | 86.63 | 86.63 | 84.68 | 83.57 | 81.34 |
| **oxyR** | 67.19 | 96.11 | 94.56 | 93 | 93 |
| **dksA** | 90.32 | 90.32 | 84.59 | 88.17 | 88.17 |
| **nsrR** | 100 | 99.72 | 91.17 | 91.17 | 91.17 |
| **dnr** | 0 | 0 | 11.57 | 0 | 0 |
| **ompR** | 92.18 | 90.74 | 92.18 | 92.39 | 92.39 |
| **baeR** | 35.08 | 26.65 | 35.31 | 35.31 | 35.31 |
| **baeS** | 0 | 0 | 0 | 0 | 0 |
| **gmk** | 100 | 94.09 | 98.25 | 99.34 | 99.34 |
| **rcsB** | 99.77 | 68.11 | 89.75 | 82.69 | 82.92 |
| **rcsC** | 0 | 0 | 0 | 0 | 0 |
| **rpoE** | 90.27 | 89.03 | 85.04 | 84.54 | 84.54 |
| **rseA** | 60.23 | 27.67 | 37.44 | 57.67 | 57.91 |
| **degS** | 94.42 | 73.82 | 91.32 | 91.56 | 91.56 |
| **recA** | 82.15 | 82.15 | 78.61 | 81.16 | 81.16 |
| **lexA** | 99.77 | 51.74 | 93.97 | 96.29 | 96.29 |
| **rpoS** | 82 | 82 | 81.21 | 78.98 | 79.24 |
| **fruR** | 85.03 | 85.03 | 84.41 | 83.49 | 83.95 |
| **spoT** | 98.48 | 98.48 | 95.38 | 96.17 | 96.17 |
| **fur** | 71.22 | 71.22 | 68.6 | 67.73 | 67.73 |
| **dps** | 48.98 | 88.63 | 89.21 | 88.63 | 89.21 |
| **fnr** | 0 | 0 | 11.57 | 0 | 0 |
| **rpoA** | 88.87 | 88.87 | 88.57 | 88.72 | 88.72 |
| **h-ns** | 96.34 | 96.34 | 66.49 | 13.93 | 13.93 |
| **katA** | 0 | 0 | 0 | 0 | 0 |
| **envZ** | 92.93 | 92.93 | 91.09 | 90.54 | 90.43 |
| **ompR** | 92.93 | 92.93 | 91.09 | 90.54 | 90.43 |
| **nlpD** | 78.34 | 73.83 | 75.09 | 75.09 | 75.09 |
| **degQ** | 94.42 | 73.82 | 91.32 | 91.56 | 91.56 |
| **recX** | 97.16 | 35.65 | 94.64 | 93.06 | 93.06 |
| **rseB** | 90.98 | 49.34 | 65.92 | 91.64 | 92.18 |

**Table S4.** Bacterial secretion systems of *R. solanacearum*

| Sl. No. | Secretion system | Gene | KO No. |
|---|---|---|---|
| 1 | Tat | TatA/ TatA/E | K00832 |
| 2 | T1SS | LipA/LipC | K01046 |
| 3 | T1SS | CyaB | K01768 |
| 4 | T5SS | AspA | K01744 |
| 5 | T4SS | Alr | K01775 |
| 6 | T1SS | HlyD | K01993 |
| 7 | T1SS | HlyD | K02005 |
| 8 | T1SS | HlyD | K02022 |
| 9 | T1SS | ABC transporter | K02056 |
| 10 | T1SS | ABC transporter | K02057 |
| 11 | T1SS | ABC transporter | K02058 |
| 12 | Tad | Flp/ Fap/ PilA/ CpaB | K02279 |
| 13 | T3SS | CpaC/RcpA | K02280 |
| 14 | T4P | PilV/ CpaD | K02281 |
| 15 | Tad | TadZ/CpaE | K02282 |
| 16 | Tad | TadA/CpaF | K02283 |
| 17 | T4SS | Alr | K02314 |
| 18 | Flg | FlGB | K02387 |
| 19 | Flg | FlGC | K02388 |
| 20 | T3SS | FlhB | K02401 |
| 21 | Flg | FliE | K02408 |
| 22 | T3SS | FliH | K02411 |
| 23 | T2SS | GspN/XcpP | K02452 |
| 24 | T2SS | GspD | K02453 |
| 25 | T2SS | GspE/HxcR/XcpR/PilB | K02454 |
| 26 | T2SS | GspF/PilC/XcpS | K02455 |
| 27 | T2SS | GspG/XcpT | K02456 |
| 28 | T2SS | GspH/XcpU | K02457 |
| 29 | T2SS | GspI/XcpV | K02458 |
| 30 | T2SS | GspJ/XcpW | K02459 |
| 31 | T2SS | GspK/XcpX | K02460 |
| 32 | T2SS | GspL/XcpY | K02461 |
| 33 | T2SS | GspM/XcpZ | K02462 |
| 34 | T2SS | GspN | K02463 |
| 35 | T4P | PilA/Flp | K02650 |
| 36 | T4P | PilA/Flp | K02651 |
| 37 | T4P | PilB/ GspE | K02652 |
| 38 | T2SS | GspF/PilC/XcpS | K02653 |
| 39 | T2SS | GspO | K02654 |
| 40 | T4P | PilN | K02663 |
| 41 | T4P | PilT | K02669 |
| 42 | T4P | PilU | K02670 |
| 43 | T9SS | SprT | K02742 |
| 44 | Sec-SRP | SecA | K03070 |

| | | | |
|---|---|---|---|
| 45 | Sec-SRP | SecB | K03071 |
| 46 | Sec-SRP | SecD | K03072 |
| 47 | Sec-SRP | SecE | K03073 |
| 48 | Sec-SRP | SecF | K03074 |
| 49 | Sec-SRP | SecG | K03075 |
| 50 | Sec-SRP | SecY | K03076 |
| 51 | T5SS | LepB/IepB | K03100 |
| 52 | Sec-SRP | Ffh/Srp54 | K03106 |
| 53 | Sec-SRP | FtsY | K03110 |
| 54 | T2SS | DamX | K03112 |
| 55 | Tat | Tat A | K03116 |
| 56 | Tat | TatB | K03117 |
| 57 | Tat | TatC | K03118 |
| 58 | T4SS | VirB1/ AvhB1 | K03194 |
| 59 | T4SS | VirB10/ AvhB10 | K03195 |
| 60 | T4SS | VirB11/ AvhB11 | K03196 |
| 61 | T4SS | VirB2/ AvhB2 | K03197 |
| 62 | T4SS | VirB3/ AvhB3 | K03198 |
| 63 | T4SS | VirB4/ AvhB4 | K03199 |
| 64 | T4SS | VirB5/ AvhB5 | K03200 |
| 65 | T4SS | VirB6/ AvhB6 | K03201 |
| 66 | T4SS | VirB7/ AvhB7 | K03202 |
| 67 | T4SS | VirB8/  AvhB8 | K03203 |
| 68 | T4SS | VirB9/ AvhB9 | K03204 |
| 69 | Sec-SRP | YajC | K03210 |
| 70 | Sec-SRP | YidC/Oxa1 | K03217 |
| 71 | T3SS | PscC/YscC/EscC/HrcC | K03219 |
| 72 | T3SS | PscD | K03220 |
| 73 | T3SS | PscF | K03221 |
| 74 | T3SS | PscJ/YscJ/EscJ/HrcJ | K03222 |
| 75 | T3SS | PscL | K03223 |
| 76 | T3SS | PscN/YscN/EscN/HrcN/FilI | K03224 |
| 77 | T3SS | PscQ/RhcQ | K03225 |
| 78 | T3SS | PscR/YscR/EscR/HcrN | K03226 |
| 79 | T3SS | YscS/EscS/HcrS | K03227 |
| 80 | T3SS | PscT/YscT/EscT/HcrT | K03228 |
| 81 | T3SS | PscU/YscU/EscU/HcrU | K03229 |
| 82 | T3SS | PscV/RhcV/PcrD | K03230 |
| 83 | T4SS | TraG/ VirD4 | K03250 |
| 84 | Tat | TatD/MttC | K03424 |
| 85 | T1SS | HlyD | K03543 |
| 86 | T5SS | LepA | K03596 |
| 87 | T5SS | SspA | K03599 |
| 88 | T5SS | SspB | K03600 |
| 89 | T4SS | MobB | K03753 |
| 90 | T3SS | PscB | K04049 |
| 91 | T3SS | PscE | K04050 |
| 92 | T3SS | PscG | K04051 |

| | | | |
|---|---|---|---|
| 93 | T3SS | PscH | K04052 |
| 94 | T3SS | PscI | K04053 |
| 95 | T3SS | PscK | K04054 |
| 96 | T3SS | PspO | K04056 |
| 97 | T3SS | PscP | K04057 |
| 98 | T3SS | PscW/PopN | K04058 |
| 99 | T3SS | PscX | K04059 |
| 100 | T3SS | PscY | K04060 |
| 101 | T3SS | FlhB | K04061 |
| 102 | T6SS | ImpA/ OstA/ LptA | K04744 |
| 103 | T1SS | HlyB/ CyaB/ MsbA | K06147 |
| 104 | T1SS | HlyB/ CyaB/ MsbA | K06148 |
| 105 | T4SS | TraR/DksA/DnaK | K06204 |
| 106 | Tat | TatE | K06886 |
| 107 | T6SS | HcpA | K07126 |
| 108 | T4SS | TrbL | K07344 |
| 109 | T5SS | AlpA | K07733 |
| 110 | T1SS | HlyD | K07798 |
| 111 | T6SS | ImpA/ OstA/ LptA | K09774 |
| 112 | T1SS | HlyD | K11003 |
| 113 | T1SS | HlyB/ CyaB/ MsbA | K11004 |
| 114 | T1SS | MsbA | K11085 |
| 115 | T6SS | ImpM/TssM | K11890 |
| 116 | T6SS | ImpL/TssL | K11891 |
| 117 | T6SS | ImpK/TssK/DotU | K11892 |
| 118 | T6SS | ImpJ/TssJ | K11893 |
| 119 | T6SS | ImpI/TssI | K11894 |
| 120 | T6SS | ImpH/TssH | K11895 |
| 121 | T6SS | ImpG/TssG | K11896 |
| 122 | T6SS | ImpF/TssF | K11897 |
| 123 | T6SS | ImpC/TssC | K11900 |
| 124 | T6SS | ImpB/TssB | K11901 |
| 125 | T6SS | HcpA | K11902 |
| 126 | T6SS | Hcp | K11903 |
| 127 | T6SS | ImpA/VgrG/VasJ/TssA | K11904 |
| 128 | T6SS | VasG | K11907 |
| 129 | T6SS | ImpA/VgrG/VasJ/TssA | K11910 |
| 130 | T1SS | TolC | K12340 |
| 131 | Tad | TadB | K12510 |
| 132 | Tad | TadC | K12511 |
| 133 | T1SS | AprD/HasD | K12536 |
| 134 | T1SS | AprE | K12537 |
| 135 | T1SS | AprF | K12538 |
| 136 | T1SS | HlyD | K12542 |
| 137 | T1SS | TolC | K12543 |
| 138 | T1SS | AprA/HasA | K12545 |
| 139 | T4SS | TraI/LasI | K13060 |
| 140 | T5SS | IscA | K13628 |

| | | | |
|---|---|---|---|
| 141 | T1SS | HlyD | K15549 |
| 142 | T1SS | HlyD | K15727 |
| 143 | T1SS | HlyD | K18901 |
| 144 | T4SS | TraR/ DksA | K19732 |
| 145 | T4SS | TrbJ | K20266 |
| 146 | T4SS | TrbB | K20527 |
| 147 | T4SS | TrbC | K20528 |
| 148 | T4SS | TrbD | K20529 |
| 149 | T4SS | TrbE | K20530 |
| 150 | T4SS | TrbF | K20531 |
| 151 | T4SS | TrbG | K20532 |
| 152 | T4SS | TrbI | K20533 |
| 153 | T3SS | RetS | K20972 |
| 154 | T1SS | HlyD | K21136 |

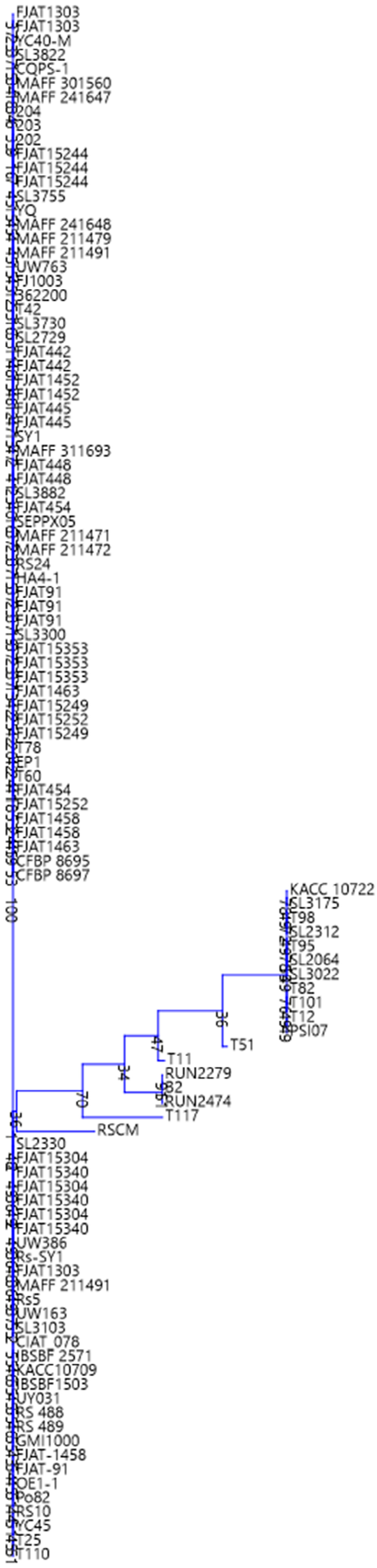


**Figure S1.** Cluster dendrogram based on distribution of stress responsive genes within *R. solanacerium* species complex.
